# Educational and programmatic components of effective school-based food and nutrition education: an umbrella review

**DOI:** 10.1017/S1368980026103024

**Published:** 2026-07-03

**Authors:** Natalie H. Greaves-Peters, Pamela Ann Koch, Randi Wolf, Jennifer W. Cadenhead

**Affiliations:** 1 Health Studies & Applied Educational Psychology, Teachers College at Columbia University, USA; 2 Department of Health and Behavior Studies, Columbia University, USA

**Keywords:** School-based food and nutrition education, Food and nutrition education programs, Dietary behaviour change, Educational and programmatic components, K-12 nutrition education

## Abstract

**Objective:**

To identify and synthesise the educational and programmatic components most consistently associated with effective school-based food and nutrition education (SBFNE) for children and adolescents.

**Design:**

An umbrella review was conducted in accordance with PRISMA 2020 guidelines. We systematically searched CLEO, EDUCAT+, PubMed and Google Scholar through December 2022 for eligible literature reviews and meta-analyses. Included reviews were assessed for methodological quality and synthesised at the component level using qualitative and quantitative approaches.

**Setting:**

Reviews examined SBFNE interventions delivered primarily during regular school hours in primary and secondary education settings worldwide.

**Results:**

Forty-four literature reviews synthesising evidence from 1651 primary studies (1115 unique) were included. Across reviews, twenty distinct educational and programmatic components were identified and standardised. Components most frequently reported and consistently associated with positive dietary behaviour change included *Family Engagement*, *Focused on Behaviour Change*, *Multicomponent Approaches* and *Integration into the School Curriculum*. Other components – such as *Cultural Inclusivity* and *Environmental Sustainability* – were examined less frequently but were associated with positive dietary outcomes in the reviews that assessed them. Several components, including *Peer Support*, *Technology-Based Engagement* and *Community Engagement*, demonstrated mixed or context-dependent effects, reflecting variability in implementation and study design. No component was consistently associated with adverse dietary outcomes.

**Conclusions:**

By shifting the analytic focus from individual interventions to cross-cutting components, this umbrella review clarifies which elements most consistently underpin effective SBFNE. The resulting component framework provides a practical, transferable foundation to inform program design, evaluation and policy decision-making and to strengthen the equity, coherence and effectiveness of SBFNE initiatives.

## Rationale

Numerous reviews have examined school-based food and nutrition education (SBFNE) across diverse educational contexts^(1–44)^. Collectively, this body of work reflects growing concern about poor diet quality among children and adolescents and its contribution to diet-related chronic diseases worldwide. As nutrition science and food environments have evolved, SBFNE has increasingly shifted from a sole emphasis on information dissemination towards approaches that prioritise dietary behaviour change, reflecting broader shifts in public health and education priorities^(2,45–49)^.

Poor dietary behaviours among children – characterized by low fruit and vegetable intake and high consumption of energy-dense, nutrient-poor and highly processed foods – contribute to rising rates of obesity and other chronic conditions later in life^(47–49)^. These dietary behaviours are shaped early in life and are further reinforced by food environments that disproportionately expose children, particularly those in underserved communities, to aggressive marketing of unhealthy food products^(50,51)^.Schools represent a critical setting for early intervention, given their reach, structure and potential to influence dietary behaviours across developmental stages.

SBFNE programs aim to equip children and adolescents with the knowledge, skills and decision-making capacities needed to develop healthier eating behaviours^(52)^. However, despite a substantial body of review-level evidence, findings are dispersed across diverse intervention types, populations and study designs. As a result, the educational and programmatic components associated with effectiveness are often reported as part of bundled, multilevel interventions rather than examined individually, limiting cross-study comparison^(4,32)^.

Given the increasing complexity of modern food environments and the growing interest in scaling effective school-based strategies, there is a pressing need to move beyond individual interventions and identify the cross-cutting components that underpin successful SBFNE programs. This umbrella review addresses this gap by synthesising evidence across existing literature reviews to identify educational and programmatic components associated with effective SBFNE. By consolidating findings across diverse intervention contexts, this study provides a component-level perspective on what contributes to successful SBFNE implementation.

## Objectives

This umbrella review seeks to systematically identify and evaluate the educational and programmatic components that contribute to the effectiveness of SBFNE programs delivered to children and adolescents in primary and secondary education settings worldwide. By synthesising findings from existing literature reviews, this study aims to clarify which components are most consistently associated with positive dietary behaviour change and to inform future research, practice and policy.

## Methods

### Eligibility criteria

We *included* literature reviews and meta-analyses examining SBFNE programs delivered primarily during regular school hours to students enrolled in primary and secondary education (approximately ages 5–18 years) in public, private or charter schools worldwide. Eligible reviews examined programs integrated into the general school curriculum or school environment with the aim of promoting healthier dietary behaviours.

Included interventions encompassed classroom-based nutrition curricula; experiential learning approaches (e.g. school gardens, cooking or culinary education); food and nutrition literacy interventions; multicomponent programs combining education with environmental or policy strategies and programs incorporating family or community engagement. Reviews were eligible if interventions were delivered within the regular school day and evaluated students on at least one dietary or diet-related behavioural outcome, regardless of whether the intervention also targeted nutrition knowledge, food literacy skills or dietary decision-making.

Outcomes of interest included changes in fruit and vegetable intake and/or reductions in consumption of foods described across reviews using varied terminology, including ultra-processed foods, highly processed foods, foods high in fat, sugar and salt, snack foods, junk food, sugar-sweetened beverages, soda or vending machine offerings; terminology varied across reviews, reflecting shifts in nutrition science and measurement over time. Reviews were eligible regardless of whether reported effects were positive, null, negative or mixed and were not excluded based on statistical significance. Instead, outcomes were extracted and categorised to characterise the direction and consistency of reported effects across reviews.

We defined a *component* as a distinct educational or programmatic feature considered essential to the design, implementation, sustainability or evaluation of a primary or secondary (K-12) SBFNE program. *Educational components* refer to instructional content and pedagogical strategies intended to influence student knowledge, skills, attitudes or dietary behaviours. *Programmatic components* refer to structural, organisational, environmental or implementation-related features that support, enable or sustain the delivery of food and nutrition education within school settings. Only English-language reviews published in peer-reviewed journals, government reports or accredited academic institutions were included.

We *excluded* reviews that focused exclusively on specialised populations (e.g. children with clinical conditions or specific dietary needs), evaluated non-food-based interventions, targeted activities conducted primarily outside regular school hours, were unpublished, or non-peer-reviewed or reported in languages other than English. Full inclusion and exclusion criteria are provided in Table 1.

**Table 1.** Umbrella review inclusion/exclusion criteria Table 1 long description.

Criteria	Inclusion	Exclusion
1	Reviews of studies/Review articles	Not a review of studies/review article
2	Reviews of studies that included SBFNE as a component of the program	Reviews of studies that did not include a SBFNE intervention component
3	Reviews of studies that focused on increasing fruits and vegetables intake and pinpointed at least one intervention strategy that contributed to making the SBFNE related intervention effective.	Reviews of studies that did not identify any intervention strategies that contributed to the program’s effectiveness
4	Reviews of studies that included students in grades K-12 or primary or secondary school (typically between the ages of 5 and 18 years)	Reviews of studies that included persons outside of grades K-12, or primary or secondary school
5	Reviews of studies that were implemented at a public, private or charter school anywhere in the world during regular school hours	Reviews of studies that were only implemented outside of regular school hours
6	Reviews of studies that were geared at primary prevention for dietary behaviour change that promotes personal health	Reviews of studies that exclusively evaluated school-based interventions as treatment for a specific disease or condition such as overweight and obesity and non-food-based approaches (e.g. use of supplements)
7	Reviews of studies that focused on general population, as opposed to, for e.g. overweight or obese children only	Reviews of studies that focused on children with special dietary needs
8	Reviews of studies that were published in English	Reviews of studies not reported in English
9	Reviews of studies published in peer reviewed journals, government reports, or reputable academic institutions from inception to present.	Reviews of studies that were non-peer-reviewed work (e.g. thesis, gray literature, unpublished reports, book chapters)

### Information sources

To identify relevant literature, we searched CLEO, EDUCAT+, PubMed and Google Scholar to capture educational, health and nutrition-related research relevant to school-based interventions across diverse contexts. The final database search was on 31 December 2022. We also screened the reference lists of included reviews to identify additional eligible records. The Cochrane Database of Systematic Reviews was searched as part of the screening process; however, no Cochrane reviews met the predefined eligibility criteria.

### Search strategy

We developed the search strategy using the Population, Intervention, Comparison, Outcomes and Time (PICO[T]) framework in collaboration with a research librarian^(53)^. Search terms were designed to identify literature reviews and meta-analyses on SBFNE programs; the full strategy (including keywords and Boolean operators) is provided in Table 2. Where available, database filters were used to limit results to English-language reviews published between 1900 and 2022 and to restrict publication type to literature reviews and meta-analyses. Search terms and screening procedures were pilot-tested on a small set of records prior to full screening.

**Table 2.** PICO(T) search strategy Table 2 long description.

[P] Population	What are the characteristics of my study population? Students in grades K-12, aged approximately 5–18 years, from public, private, or charter schools globally.	‘k-12’ OR ‘kindergarten-12th grade’ OR kindergarten-12’ OR ‘elementary school*’ OR ‘middle school*’ OR ‘high school*’ OR ‘primary school*’ OR ‘secondary school*’ OR ‘school-age’ OR ‘Elementary Secondary Education’ OR ‘school-based’ OR ‘adolescents’
[I] Intervention	What intervention am I investigating? Literature reviews that primarily examined SBFNE interventions, which must be predominantly conducted during regular school hours.	‘nutrition instruction’ OR ‘nutrition education’ OR ‘cooking instructions’ OR ‘agricultural education’ OR ‘obesity prevention’ OR ‘school-based food and nutrition education’ OR ‘school-based’ OR ‘health promotion interventions’ OR ‘school gardens’ OR ‘home-grown school feeding programs’ OR ‘dietary intake’ OR ‘culinary nutrition education’
[C] Comparison intervention (or control)	What is the alternative to the intervention? Not a factor.	curricul* OR framework* OR tool* OR ‘lesson’
[O] Outcome(s) of interest	What is the relevant outcome? Only literature reviews reporting overall dietary behaviour outcomes in the general student population. All literature review types.	‘health behavior’ OR ‘health behaviour’ OR ‘behavior change’ OR ‘change strateg*’ OR ‘habit formation’ OR ‘behavior pattern*’ OR ‘behaviour pattern*’ OR ‘nutrition-related knowledge’ OR ‘attitudes and practices’ OR ‘dietary and nutrition-related outcomes’
[T] Time period	When did the study occur? From the year 1900 to present.	‘1900 to present’

### Screening and selection process

We used a two-step screening approach. The primary reviewer (NGP) screened titles and abstracts for relevance and retrieved full-text for records deemed relevant or unclear. A second reviewer (PAK) independently verified the inclusion and exclusion decisions at the full-text stage. Disagreements were resolved through discussion to reach a consensus. No automation tools were used. Screening decisions and reasons for exclusion were documented using a standardised screening template.

### Data collection process

Data were extracted using a standardised template based on the PICO(T) framework. The template was pilot-tested on three reviews and refined prior to full extraction. Extracted data were used to support component-level quantitative and qualitative synthesis, as well as descriptive assessment of review-level characteristics. The final data extraction template is provided in the online Supplementary Material.

To support reliability, both reviewers independently extracted data for the first ten reviews. For the remaining reviews, NGP conducted extraction and PAK verified accuracy and consistency. Ambiguities or discrepancies (e.g. differences in how reviews operationalised *Family Engagement v*. *Multicomponent Approach*, evolving terminology such as *‘experiential learning’* or limited outcome reporting in lower-quality reviews) were resolved through discussion to ensure consistent coding and interpretation.

### Data items

Data items extracted from each included review included population characteristics, intervention type and setting, dietary and diet-related behavioural outcomes (as defined in the eligibility criteria) and the educational or programmatic components explicitly linked to those outcomes. We also extracted review-level methodological characteristics (e.g. design, conduct and quality appraisal methods) and reported funding sources to assess potential conflicts of interest.

To identify programme components, we examined all included reviews and generated an initial components set based on recurring themes, which was iteratively refined through comparison across the full review sample. Component definitions were standardised to improve coherence and reduce redundancy while preserving meaning as reported in the source reviews. As part of this process, an AI-assisted language tool (ChatGPT) was used to flag unclear phrasing and redundancies in draft component definitions; all suggested edits were reviewed by the research team and incorporated only when consistent with the original literature^(54)^. Final component definitions were confirmed through reviewer consensus.

### Study risk of bias assessment

We assessed the methodological quality of included reviews using a structured quality appraisal framework that combined Snyder’s guidelines with additional SBFNE-specific criteria. Snyder’s framework contributed twenty-one core criteria across four phases: *Design* (Criteria 1–6), *Conduct* (8–12), *Data Abstraction and Analysis* (15–19) and *Structuring and Writing* (20–24)^(55)^. To ensure relevance to SBFNE, we supplemented these with additional criteria assessing the use of psychosocial theory and evidence-based educational strategies. In total, twenty-four criteria were applied. The quality assessment template is provided in the online Supplementary Material.

### Reporting bias assessment

We assessed potential reporting bias by examining whether the included reviews transparently reported intended outcomes or selectively emphasized favourable findings^(55,56)^. We also considered publication bias, recognising that reliance on published studies may overrepresent positive findings and examined discrepancies between the stated objectives and reported outcomes^(55,56)^. As this was a qualitative synthesis, we did not apply formal statistical tests for reporting bias; instead, we conducted a narrative assessment of how missing or selectively reported results might influence interpretation^(55,57)^.

### Effect measures

Given that most included reviews did not report pooled effect estimates, we conducted a qualitative synthesis based on review authors’ reported associations between components and dietary behaviour outcomes. Reported effects were categorised as positive, negative, no effect or mixed based on the direction and consistency of outcomes described within each review:

- **Positive effect:** improved dietary behaviour (e.g. increased fruit and vegetable intake and/or reduced consumption of foods described as highly processed/unhealthy).
- **Negative effect:** adverse dietary changes or unintended consequences counteracting intended behaviour change goals.
- **No effect:** no measurable changes in dietary behaviours.
- **Mixed effect:** inconsistent findings across included primary studies (e.g. a combination of positive, null or negative results).

These classifications were applied consistently across reviews during synthesis to characterise patterns of effects rather than to determine study eligibility.

### Synthesis methods

We synthesised findings by identifying and standardising educational and programmatic components and summarising their prevalence and reported direction of effect across included reviews. Using thematic analysis, we compared component descriptions and reported outcomes across reviews and tabulated results to summarise prevalence and effectiveness^(57)^. Visual diagrams were developed to illustrate component frequency and trends.

To explore heterogeneity, we examined whether reported effectiveness varied across program characteristics (e.g. setting, target population and programme duration) and whether patterns differed by review quality or reporting standards. A sensitivity analysis restricted the synthesis to high-quality reviews to assess robustness of the findings^(55,57)^.

### Certainty assessment

Following the Preferred Reporting Items for Systematic Reviews and Meta-Analyses (PRISMA) 2020 guidelines, we assessed certainty of evidence by considering the consistency with which components were reported across the included reviews and alignment in reported direction of effects^(58,59)^. Components frequently reported across multiple reviews with consistent findings were interpreted as having stronger supporting evidence, whereas components with limited representation or inconsistent findings were interpreted with greater caution.

As detailed above, we also conducted a structured quality evaluation of the included reviews. Reviews meeting at least ≥ 21 of the twenty-four criteria were classified as high quality, those meeting 18–20 as medium quality and those meeting < 18 as low quality. These thresholds align with established practices in systematic reviews, where predefined cut-offs enhance reproducibility and comparability^(60)^. Consistent with the GRADE framework, we interpreted certainty along a spectrum; to align with our predefined quality thresholds, we consolidated ‘low’ and ‘very low’ into a single ‘low’ category to maintain consistency with our predetermined quality assessment criteria^(58,60)^.The primary reviewer (NGP) conducted quality assessment and the second reviewer (PAK) independently verified them; discrepancies were resolved through discussion.

## Results

### Study selection

**Figure 1.**
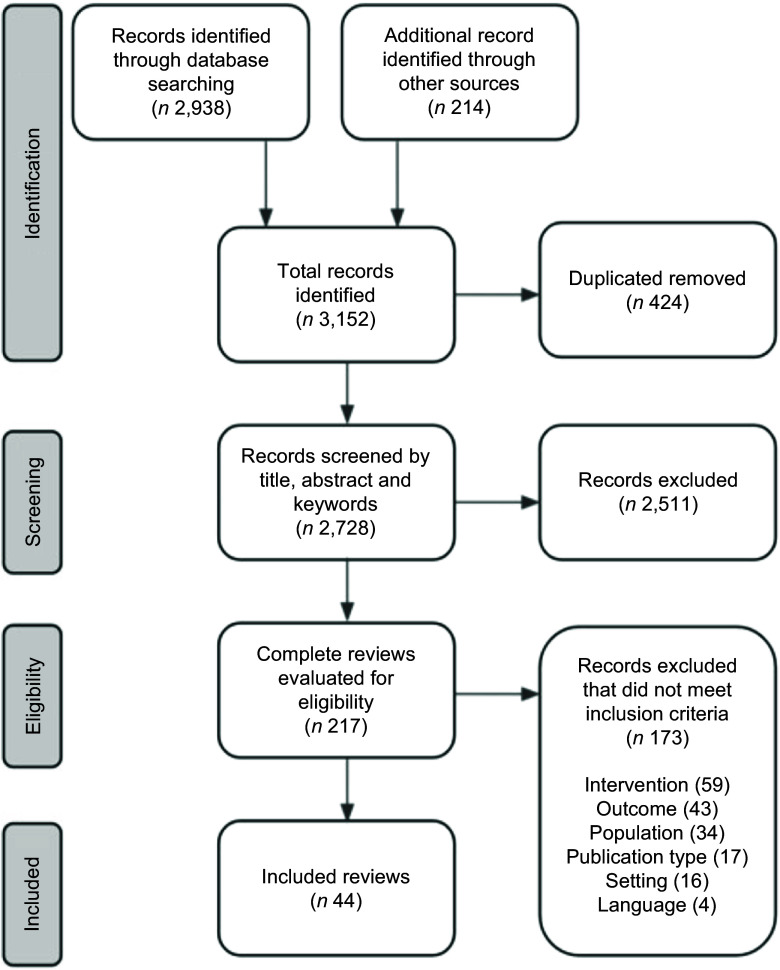
Figure 1 long description. PRISMA flow diagram.

The study selection process is summarised in the PRISMA flow diagram (Figure 1)^(59)^. An initial search across selected databases yielded 2938 records, with an additional 214 records identified through reference list screening and other sources, resulting in 3152 total records. After removing 424 duplicates, 2728 records were screened by titles, abstracts and keywords. Of these, 2511 records were excluded for not meeting eligibility criteria. A total of 217 records underwent full-text review. Following this stage, 173 records were excluded for reasons including inappropriate intervention type (*n* 59), outcomes not aligned with the study objectives (43), unsuitable population (34), incorrect publication type (17), setting mismatch (16) and language barriers (4). Ultimately, forty-four literature reviews were included in this umbrella review^(1–44)^.

### Study characteristics

The forty-four included literature reviews, published between 1959 and 2022, are summarised chronologically in Table 3. Although the search window extended from 1900 to 2022, no eligible reviews were published prior to 1959. Together, these reviews synthesise evidence from forty-six countries across six continents, reflecting a broad geographic scope and increasing international representation over time^(1–44)^. Across reviews, SBFNE programs most commonly incorporated curriculum-based instruction, experiential learning approaches (e.g. gardening or cooking) and multicomponent strategies integrating educational, environmental and family-engagement elements.

**Table 3.** Characteristics of the 44 included literature reviews Table 3 long description.

Citation	Year	Search window	Country of primary research	Study setting	Primary study designs	Review type	Stated aim of review	Inclusion criteria	Num^*^
Jacobson *et al.* ^(1)^	1959	Not cited	Canada, U.S.A.	In the classroom; within community settings	Classroom observations	Report	Identify promising practices in nutrition education in elementary schools and explore ways to improve children’s nutrition.	Elementary school	14
Whitehead^(2)^	1973	1900–1970	Canada, Colombia, India, Mexico, Panama, Philippines, South Africa, Thailand, Turkey, U.S.A., Venezuela, Vietnam	In the classroom; public health clinics; university; professional schools	Mixed studies: Indexes, abstracts, journals, theses, books, periodicals	Literature review	Compile a comprehensive bibliography of SBFNE studies conducted between 1900 and 1970, identify challenges and make recommendations for effective nutrition education.	The primary focus of the review was on SBFNE, although children and adult programs in public health clinics, university and professional schools were also examined.	268
White- Stevens & NJ State DOE^(3)^	1981	1979–1980	U.S.A. (New Jersey only)	In the classroom	Project descriptions	Report	Describe all projects funded by the Nutrition Education and Training Program of the New Jersey State Department of Education during 1979 and 1980 that focus on enhancing nutrition knowledge and promoting nutrition practices, behaviours and attitudes among students, parents and the community.	Nutrition education and training programs funded by New Jersey State Department of Education (1979–1980). Scope: Broad, focusing on children, with emphasis on primary school students.	232
Contento^(4)^	1992	1978–1988	U.S.A.	School-based	RCT, non-RCT	Review	Assess the current state of knowledge regarding general nutrition education, targeted behaviour change, teacher preparation, parental involvement and food service involvement. Also, the study aims to examine research design and methodological issues and identify implications for research and practice in the field.	Peer-reviewed journal publications focusing on classroom-based interventions delivered by teachers or researchers. Study design had to include both comparison and treatment groups, while examining behavioural outcomes and other variables. Included studies also provided sufficient measurement data to evaluate the variables of interest.	17
Lytle^(5)^	1995	1980–1994	U.S.A.	Classroom; Community (grocery stores, libraries, summer youth nutrition education camp, after-school day care)	Primary intervention studies	Literature review for the USDA	Assess the effectiveness of nutrition education for school-aged children and identify additional information needed to develop nutrition education policies and effective programs.	The nutrition education program was delivered to children from kindergarten through high school ages, within or outside of a school setting. The research design included a control group. An outcome evaluation (knowledge, attitudes, or behaviour) was reported. The nutrition education program had a prevention focus and was not directed at high-risk youth.	17
Hoelscher *et al.* ^(6)^	2002	1994–2000	Australia, Taiwan, U.K., U.S.A.	In the classroom; community centres	Primary intervention studies	Systematic review	Discuss nutrition interventions during adolescence and their potential impact on children’s dietary behaviours, both in the short term and later in life. Highlight successful program characteristics and trends that can influence the development of effective nutrition programs for adolescents.	Studies that focused on adolescents aged 11–18 years (students enrolled in grades 5 through 12), were population based and conducted in schools, clinics or communities, were published between 1994 and 2000 and included a student outcome evaluation.	17
Woolfe & Stockley^(7)^	2005	2000–2002	U.K.	In primary and secondary schools	Published and grey literature	Literature review	Assess the feasibility and effectiveness of dietary change interventions implemented in U.K. school-based settings.	The studies must test the feasibility and effectiveness of dietary change interventions in U.K. primary and secondary school-based settings, and they must be final reports commissioned by the Food Standards Agency.	5
Doak *et al.* ^(8)^	2006	Up to August 2005	Australia, Chile, Germany, Greece, Israel, Italy, Russia, U.K., U.S.A.	School-based; after school; Community- based	CT, RCT	Mixed studies review/ mixed methods review	Identify successful aspects of childhood overweight prevention programs through assessing existing interventions quantitatively and qualitatively, evaluating efficacy, effectiveness, implementation and potential adverse effects.	Focus on school-based studies (age 6–19 years of age). Anthropometric measurements of body weight or adiposity (e.g. BMI or skinfolds) taken at baseline and follow-up. Intervention targeting diet or physical activity-related behaviour or both. Study monitored and evaluated with documented evidence (e.g. published paper or publicly accessible documentation).	25
Knai *et al.* ^(9)^	2006	Up to April 2004	Ireland, U.K., U.S.A.	Population-based studies (e.g. largescale FV promotions, nutrition education, social marketing approaches); individual- focused studies (e.g. small-scale and large-scale intervention studies)	RCT, non-RCT	Systematic review	Synthesise the evidence on interventions aimed at increasing fruit and vegetable consumption among children. Assess the effectiveness of various interventions and provide recommendations for future research, highlighting the strongest evidence in favour of multicomponent interventions.	Intervention Type: Individual- and population-based interventions and promotion programs targeting increased fruit and/or vegetable consumption in children. Participant Criteria: Free-living, not acutely ill children of both genders. Follow-up Period: Studies with a minimum follow-up period of 3 months. Outcome Measurement: Studies measuring change in fruit and vegetable intake and including a control group. Language: Papers published in any of the eight considered languages.	15
Sharma^(10)^	2006	1999–2004	U.K., U.S.A.	Classroom- based; school-based	Primary research of varying types, including RCT and non-RCT	Review	Review school-based obesity prevention interventions targeting childhood and adolescence, including dietary behaviour outcomes (e.g. food intake, food choice) alongside anthropometric measures.	Explicit school-based curriculum for obesity prevention. Focus on the general population rather than overweight or obese children. Study conducted in the U.S.A. or U.K. Publication in English language.	11
Peterson & Fox^(11)^	2007	1966–2001	Australia, Greece, Israel, Thailand, U.S.A.	Curriculum-based	RCT, non-RCT	Review	Create a valuable resource that can aid in the development and execution of future school-based obesity prevention interventions by a concise review of the effectiveness of existing school-based interventions, offering suggestions for enhancements based on current evidence, related research findings and expert recommendations.	Published in English. Primary interventions implemented in schools and focused on diet, physical activity or a combination of both. Studies included weight-related measures as outcomes, such as BMI, body weight and/or other anthropometric measures. Interventions did not necessarily need to focus specifically on obesity. Study design included a control measurement, either within groups (baseline *v*. follow-up) or between groups (treatment group *v*. follow up). Subjects were followed for at least six months from the beginning of the intervention.	10
Contento^(12)^ (Chapter 4 ‘Based on Lytle,1995’^(5)^	2008	1980–2007	U.S.A.	Primarily in the school setting; some were conducted outside of school	Primary intervention studies	Review of Nutrition Education Research in the Journal of Nutrition Education and Behaviour	Chapter 4 aims to identify the key elements that contribute to the effectiveness of nutrition education for school-aged children, that strives to improve children’s eating practices through behavioural change and provide conclusions on these elements.	This chapter includes studies of nutrition education targeting the general school-aged population, conducted primarily in the school setting, but some outside of school. Interventions with children who have special needs or medical conditions that require special education or counselling were excluded.	43
Van Cauwenberhe *et al.* ^(13)^	2010	1990–2007	Belgium, Denmark, France, Italy, Netherlands, Norway, Spain, Sweden, U.K.	Classroom-based; environmental modifications	Before-After, non-RCT, prospective cohort, cluster RCT	Systematic review	Provide a summary of the effectiveness of school-based interventions in promoting a healthy diet among children (6–12 years old) and adolescents (13–18 years old) by examining existing published and grey literature from Europe.	Studies evaluating school-based interventions promoting a healthy diet in children (6–12 years old) and adolescents (13–18 years old) aiming at primary prevention of obesity. Reported effects on dietary behaviour or anthropometrics.	42
AlMarzooqi & Nagy^(14)^	2011	2004–2009	Australia, Canada, China, New Zealand, U.K., U.S.A.	School; community; home	Pre/post, quasi experimental	Systematic review	Identify the theoretical frameworks that drive childhood obesity prevention programs and to identify the successful components of preventive intervention programs for childhood obesity.	Obesity intervention studies conducted in community, school and home settings targeting children and adolescents aged 6–12 years, reporting dietary behaviour outcomes alongside anthropometric measures.	22
Roseman *et al.* ^(15)^	2011	2000–2008	U.S.A.	Classroom-based	Primary interventions	Systematic review	Identify proposed recommendations for school-based nutrition interventions and evaluate the effectiveness of nutrition interventions in K-12 grade schools.	Articles published in English; intervention implemented in a school setting in the U.S.A.; being an obesity- or nutrition-related health intervention with quantitative measures; involving participants in K-12th grade.	26
Sharma^(16)^	2011	2000–2009	Chile, China, Germany, Greece, U.K., U.S.A.	Classroom- based	Quasi experimental (QE), experimental with random assignment at group level, pre-test post-test, formative evaluation	Literature review	Conduct a review of school-based interventions aimed at preventing childhood and adolescent obesity, specifically targeting the modification of dietary behaviour.	Studies published in the English language. Studies were required to focus on the general population rather than specifically targeting overweight or obese children and had an explicit dietary component in the school-based program for the prevention of obesity.	25
Evans^(17)^	2012	1985–2009	Denmark, Netherlands, New Zealand, Norway, U.K., U.S.A.	In a school setting	RCT, non-RCT	Systematic literature review	Measure the effects of school-based interventions on the consumption of fruits and vegetables in children aged 5–12 years.	Dietary intervention studies conducted in a school setting, involving children aged between 5 and 12 years at the start of the intervention. Trials with or without random assignment, including a control or usual practice group. Studies that utilised standard assessment measures such as food diaries (weighed or non-weighted methods), 24-h diet recall or FFQ were included.	27
Verstraeten^(18)^	2012	1990–2011	Brazil, Chile, China, Commonwealth of the Northern Mariana Islands (CNMI), Hungary, India, Iran, Mexico, Russia, South Africa, Thailand, Trinidad and Tobago	School-based	Peer-reviewed controlled studies, with or without randomisation	Systematic review	Conduct a systematic review of the evidence regarding the effectiveness of school-based interventions in low- and middle-income countries, targeting dietary behaviour and/or physical activity, for the primary prevention of obesity in children and adolescents aged 6–18 years.	Conducted in a school setting within a low- and middle-income country (LMIC), according to the World Bank classification; involve healthy children and adolescents aged 6–18 years; employ a controlled trial design, with or without randomisation; focus on the primary prevention of overweight or obesity through changes in dietary and/or physical activity behaviour and include both baseline and post intervention measurements of dietary and physical activity behaviour outcomes and/or anthropometric outcomes. Studies targeting parental or teacher behaviour were also eligible if outcome data were extractable for children and/or adolescents. Studies could be published in English, Spanish, French, German or Dutch.	25
Hackman & Knowlden^(19)^	2014	2003–2014	Australia, Canada, England, Greece, Iran, Scotland, South Africa, U.S.A.	School-based; home-based; community-based; multi-setting	RCT, quasi- experimental	Systematic review	Conduct a systematic review and synthesis of dietary behaviour interventions for adolescents and young adults based on the theory of planned behaviour (TPB) and theory of reasoned action (TRA).	Interventions classified as primary or secondary; utilising any quantitative design; published in the English language; targeting adolescents or young adults; having dietary change behaviour as the outcome and employing the TPB or TRA.	11
Rush & Knowlden^(20)^	2014	2003–2013	U.K., U.S.A.	School-based; community-based	Controlled trials	Systematic review	Systematically analyse interventions, conducted in community and school settings, with the objective of increasing fruit and vegetable intake (FVI) among minority children.	Quantitative preventative studies published in English language, conducted globally, targeting both antecedents to FVI as well as actual FVI. The interventions had to be multicomponent and specifically focused on school aged children, either in the community or school environment.	11
Dudley^(21)^	2015	1973–2011	Australia, Canada, Greece, Iceland, Ireland, Italy, Netherlands, New Zealand, Norway, Spain, Trinidad & Tobago, U.K., U.S.A.	Mainly curriculum- based	RCT, Controlled Clinical Trials, QE, Before-After Control Trial, Clinical trial	Systematic review	The aim of this review was twofold: (1) to conduct a systematic review of randomised controlled trials, quasi experimental studies and cluster-controlled trials investigating school-based teaching interventions aimed at improving the eating habits of primary school children and (2) to perform a meta-analysis to determine the overall effect of these interventions.	Study authors, year of publication, country(s) of study, funding agency, study design, dominant theoretical framework, study sample details (size, grade, mean age), intervention length, whether the intervention included physical activity or a specially resourced teacher, relevant outcome categories, statistical significance (*P* value/95 % CI) and the effect size of different teaching strategies on each outcome (Cohen’s d).	49
Shirley *et al.* ^(22)^	2015	2007–2012	U.S.A.	School-based; community- based	Experimental, quasi experimental	Critical review	Examine the trends in obesity prevention programs in a more homogeneous group of studies, focusing specifically on elementary school students in the U.S.A. and investigate the effectiveness of these programs by analysing rigorously designed experimental or quasi experimental studies that include objective measures of obesity related outcomes, such as BMI. Also, the review aims to explore parental and community involvement in the success of school-based obesity prevention programs.	Studies had to be published in English, targeting children ages 6–12, school-based interventions aimed at preventing obesity through physical activity, education and/or nutrition modification and utilised an experimental or quasi experimental study design with a control group. Studies organised by churches or community groups, and those focused on preventing diabetes or other metabolic syndromes were excluded.	12
Yip *et al.* ^(23)^	2015	2000–2013	Canada, U.S.A.	School-based	Most (12) had a control group with either no intervention of a lower dose of the intervention	Systemic review	Provide a summary of published refereed research on peer-led nutrition education programs in schools in the U.S.A. and Canada. Additionally, the study aimed to identify research gaps and offer recommendations for future research in this area.	The interventions included peer led nutrition education, conducted in a school setting (elementary or high school), investigating outcomes for school-aged youth (5–18 years old), taking place in Canada or the U.S.A. Additionally, the studies had to report quantitative results and be published in English	17
Meiklejohn *et al.* ^(24)^	2016	2000–2014	Australia, Belgium, Greece, Finland, Netherlands, Norway, Sweden, U.S.A.	Classroom- curriculum; School- environment	RCT	Systematic review	Expand and enhance the previous review conducted by Hoelscher *et al.* (2002) by investigating the effects of multi-strategy interventions, including nutrition education, on the health, nutrition outcomes and behaviours of adolescents.	RCT that aimed to evaluate multi-strategy interventions incorporating nutrition education and focused on adolescent populations (10–19 years old, per WHO) in developed countries, as defined by the World Bank, that reported relevant outcome measures related to health, nutrition or behaviour.	13
Murimi *et al.* ^(25)^	2018	2009–2016	73 % outside the U.S.A. (*n* 30), 27 % within U.S.A. (*n* 11)	Classroom	QE, RCT, cluster-RCT, Pre-post, longitudinal, randomised multiyear intervention	Systematic review	Examine and identify factors linked to successful nutrition education interventions conducted in children.	Studies published in English that involved a nutrition education intervention among children aged 2–19 years.	41
Muzaffar *et al.* ^(26)^	2018	1998–2013	Australia, England, U.S.A.	School setting	RCT, QE	Narrative Review	Evaluate culinary skill interventions in schools for children aged 5–12 years to identify effective programs and factors associated with improved diet quality, BMI and psychosocial outcomes.	Studies teaching ‘hands-on’ culinary skills as standalone or comprehensive interventions. Cooking classes conducted in the school setting. Children and adolescents with or without parental involvement. Interventions conducted with healthy populations. RCT or QE trials. Availability of quantitative or qualitative data provided by study participants. Program evaluation conducted by program staff.	6
Chaudhary *et al.* ^(27)^	2020	2014–2019	Australia, Belgium, Brazil, China, Ecuador, Germany, Greece, Israel, Italy, Lebanon, Netherlands, Portugal, South Africa, Spain, Sweden, U.K., U.S.A.	Primary and secondary schools	RCT, cluster RCT, CT, pre-/post-test with and without control (PP), experimental design (Quasi).	Systematic review	Assess the effectiveness of school-based food and nutrition interventions on health outcomes by reviewing evidence-based intervention studies among children internationally.	Studies investigating the effectiveness of school-based interventions targeting food and nutrition behaviour, healthy eating and nutrition education as the primary focus. Primary school children aged 5–14 years, inclusive of both genders and socio-economic backgrounds.	43
Chavez & Nam^(28)^	2020	2000–2017	Argentina, Brazil, Chile, Mexico, Peru	School-based	QE, non-RCT, cluster RCT, RCT, before and after	Systematic review	Assess the implementation and effectiveness of school-based interventions for obesity prevention in Latin America and offer recommendations for future prevention efforts in the region.	Articles published in English, Spanish and Portuguese that focused on obesity prevention in Latin American school-aged children and adolescents (6–18 years old) that had at least one school-based component and reported at least one obesity related outcome. Study designs including controlled or before and after designs. Studies providing information on intervention components and/or processes.	16
Cotton *et al.* ^(29)^	2020	1990–2018	Australia, Canada, Greece, Ireland, Italy, Netherlands, Norway, Portugal, Trinidad & Tobago, U.K., U.S.A.	Curriculum-based; school-based	CT, QE, RCT, meta-analysis	Updated systematic review and meta-analysis	Examine the impact of nutrition education programs on elementary-aged students’ energy intake, fruit, vegetable, sugar consumption and nutritional knowledge and determine the effectiveness of nutrition education delivered by qualified teachers in elementary schools. Then provide evidence-based insights for policy makers and pedagogues in designing effective teaching strategies.	Targeted elementary-aged children’s nutritional consumption or knowledge. Implemented a nutritional education program taught by an elementary school teacher. Reported nutritional consumption and/or knowledge outcomes using independent group difference values.	34
Dabravolskaj *et al.* ^(30)^	2020	2012–2020	Australia, Belgium, Canada, Denmark, France, Germany, Ireland, Israel, Korea, New Zealand, Norway, Spain, Sweden, Switzerland, U.K., U.S.A.	School-based	Cluster RCT, QE, pre/post-trial with a parallel, nonequivalent control group, repeated measures longitudinal study, secondary analysis, natural experiment, secondary data analysis of two cohorts	Systematic review and Meta-analysis	Assess the effectiveness of school-based intervention types, identified as feasible, acceptable and sustainable by Canadian stakeholders in health and education, for improving physical activity, fruit and vegetable intake and body weight in children.	Comparative studies evaluating school-based interventions to prevent obesity and associated risk factors (unhealthy diet, physical inactivity, sedentary behaviour) in school-aged children (4–18 years old). Studies conducted in countries with a human development index of 0·80 or greater with outcome assessments at least 6 months following the baseline assessment. Required outcome information on fruit and vegetable intake (servings or times per day), physical activity (minutes of moderate to vigorous physical activity and step counts) and/or adiposity (BMI, BMI z-score, BMI percentile, % overweight and/or obese).	83 (SR) 80 (MA)
Gonzalez- Jaramillo *et al.* ^(31)^	2020	1999–2018	Argentina, Brazil, Chile, Colombia, India, Portugal, Spain, Taiwan, Turkey, U.K., U.S.A.	School-based	QE, pre/post-test, Qualitative, CE, Observational methods, The Delphi method, mixed approach	Systematic review	Investigate the strategies used in school environments for teaching human nutrition, with a focus on multidisciplinary approaches and the inclusion of attitude dimensions. It also aimed to examine the theoretical references and didactic strategies guiding interventions for school children in addressing the issue of human nutrition. The study also aimed to identify potential difficulties and their impact on healthy lifestyles.	Studies related to the theoretical reference and the type of didactic strategies used in interventions for school children regarding human nutrition. The review also considered studies that examined the existence of multidisciplinary proposals and the inclusion of attitude dimensions. The EPPI Centre guidelines were followed during the review process.	24
Roseman *et al.* ^(32)^	2020	2009–2018	U.S.A.	School-based	Sample Selection: R (Random Sampling), C (Convenience Sampling), M (Purposive or Snowball Sampling)	Systematic review	Assess the utilization and continuity of 10 identified recommendations for school-based nutrition interventions in U.S.A. schools from 2009 to 2018 and to compare these interventions with those implemented between 2000 and 2008, with a focus on behaviour change strategies and their potential impact on reducing childhood obesity rates.	(1) The original publication must be in English. (2) The nutrition intervention should be implemented in a school setting. (3) Participants should be enrolled in grades K-12. (4) The intervention school must be in the U.S.A. (5) The first publication of the article should be between 2009 and 2018. (6) The intervention should use quantitative measures. Additional criteria for consistency with the 2000–2008 analysis include: (1) Studies focusing solely on nutrition education were included. (2) Multiple articles on the same intervention were combined. (3) Studies targeting specific clinical subpopulations were excluded. (4) Interventions solely focused on food service, vending, physical activity or before- or after-school programs were excluded.	27
Shapu *et al.* ^(33)^	2020	2013–2019	Bangladesh, Canada, China, India, Palestine, U.S.A.	School-based; Community- based	RCT, clustered RCT	Systematic review	The aim of this study was to systematically identify and summarise the characteristics of health and nutrition education interventions aimed at improving knowledge, attitudes and practices related to malnutrition among adolescents, based on previous research findings.	Adolescents aged 10–19 years with a minimum study population of 50, interventions related to nutrition, healthy eating, diet, dietary intake, anaemia and FV; studies with a comparison group without intervention or with other trial interventions; outcomes focusing on knowledge, attitude or practice; and RCT or clustered RCT, while excluding quasi-experimental studies and non-RCT or studies with unspecified design.	8
Bennett *et al.* ^(34)^	2021	Up to 31 May 2021	South Korea, U.K., U.S.A.	Classroom-based	Quasi-experimental, RCT, cluster RCT, Longitudinal comparative	Review	Provide a description of school-based experiential culinary interventions and assess their impact on child health outcomes in the 5–12 years age group.	School-based experiential culinary interventions targeting children aged 5–12 years that occurred during school hours, consist of at least three classes and have a control group.	5
Charlton *et al.* ^(35)^	2021	30 June 2020	Australia, Canada, Greece, Netherlands, South Korea, U.K., U.S.A.	Primary school settings	RCT, Quasi experimental, Cohort study	Systematic literature review	Identify the core elements of effective experiential nutrition interventions aimed at primary school children to enhance nutrition related cognitive and behavioural outcomes. This review also highlighted the main attributes of successful programs that resulted in significant improvements in the targeted outcomes, providing a comprehensive evaluation of them.	Experiential-based nutrition interventions conducted in primary school settings, involving children aged 5–12 years, and reporting in the English language.	42
Ijaz *et al.* ^(36)^	2021	Up to June 2015	Argentina, Canada, China, Denmark, France, Germany, Netherlands, New Zealand, Norway, Portugal, Sweden, U.K., U.S.A.	School-based; home-based; after school	Double blind RCT, not indicated for most studies	Realist review	Conduct a realist review that identifies and comprehends the contextual and mechanistic factors linked to the outcomes of school-based obesity prevention studies included in Brown *et al.*’s Cochrane review, with a focus on their potential implementation within U.K. primary schools.	Studies conducted in primary schools, involving children aged 4–12 years, focusing on obesity prevention interventions, and presenting mean BMI as an outcome.	24
Ismail *et al.* ^(37)^	2021	Up to August 2020	Europe (*n* 30), North America (*n* 15), Pacific (*n* 2)	School-based	RCT, controlled clinical trials, cohort trials [pre/post], cross-sectional	Systematic review and meta- analysis	Investigate the potential effectiveness of distributing fruit and vegetable (FV) as snacks with the aim of enhancing FV consumption among school-aged children.	School-aged children aged 4–14 years and interventions involving FV distribution as a snack either alone or combined with other approaches (e.g. nutrition education, parental involvement) within the school environment. The review assessed FV consumption as the outcome and considered all study designs	47 (SR) 10 (MA)
Kelly & Nash^(38)^	2021	Up 13 May 2020	Australia, Brazil, Canada, China, Cyprus, Greece, Iceland, Iran, Ireland, Italy, Netherlands, Portugal, Scotland, South Africa, Taiwan, Thailand, Trinidad & Tobago, Turkey, England, U.S.A.	Elementary school-based	RCT, QE, non-experimental design	Systematic scoping review	Address the lack of evidence on food literacy (FL) interventions in elementary schools by collating the characteristics of studies, intervention approaches, FL outcome measurements and levels of FL addressed in these interventions, to provide guidance for future interventions.	Peer-reviewed original research conducted in humans, published in English, described a program in an elementary or primary school for children aged 4–12 years and reported a preliminary (pilot or feasibility study) or outcome related to 1 of 3 FL competencies (functional, interactive and/or critical) in children.	116
Rose *et al.* ^(39)^	2021	2007–2020	Finland, France, Greece, Italy, Netherlands, Norway, Portugal, Spain, Turkey, U.K.	Mainstream, state and privately funded school settings	RCT, QE, cross- sectional	Mixed methods systematic review	Assess the effects of European school food interventions on nutrition, weight status and well-being outcomes and to investigate the experiences and perceptions of adolescents in Europe who have undergone a school food intervention or national school food policy.	Primary research studies conducted in the UK and Europe, published from 2008 onwards, examining the effect and/or experience of nutrition-focused interventions or policy approaches for young people aged 11–18 years. No restrictions on study design. All studies conducted within mainstream, state or privately funded school settings. All studies with a nutritional component, including both single and multicomponent interventions. Secondary outcomes considered were well-being and BMI.	27
Singhal *et al.* ^(40)^	2021	1974–2019	Brazil, China, Iran, Lebanon, Mexico, Thailand, Turkey	School-based	RCT, cluster-RCT	Systematic review and meta-analysis	Summarise evidence from RCT assessing the effectiveness of school-based interventions in preventing childhood obesity in middle income countries.	Middle-income country setting, randomised/cluster-randomised controlled trials, children aged 4–12 years and school-based interventions targeting dietary intake and/or physical activity.	21
Tallon *et al.* ^(41)^	2021	2000–2018	Austria, Belgium, Canada, China, Denmark, Finland, France, Germany, Greece, Sweden, Taiwan, U.K., U.S.A.	School-based	Inclusion criteria allowed all study designs, but the resulting 13 studies’ design details were not included in Table 1.	Systematic Review	Summarise evidence on the impact of technology-incorporated school-based nutrition education programs on adolescents’ acquisition of nutrition-related knowledge and behaviour change.	Studies published in English, with participants aged 12–18 years, regardless of gender, ethnic group, anthropometric classification or socio-economic status. Studies had to report a nutrition-related primary outcome (knowledge and/or behaviour) and involve school-based interventions using Information and Communication Technologies (ICT), including computers, cell phones, the internet, software, social networks and other multimedia applications and services. No restrictions were applied to study design, duration, control condition, follow-up period or type of ICT used.	13
Aceves- Martins *et al.* ^(42)^	2022	1995–2021	Mexico	School-based (89·6 %); home-based; clinic-based; community-based; leisure centres; digital domains (e.g. social media interventions)	Experimental, QE, RCT, Cohort, Controlled, pilot-RCT, pilot-cohort	Systematic review	Identify and evaluate obesity prevention interventions delivered to children and adolescents (< 18 years) in Mexico as part of the Childhood and adolescent Obesity in Mexico: Evidence, Challenges and Opportunities (COMO) project, which seeks to comprehend the extent, nature, effects and costs of childhood and adolescent obesity in Mexico.	The study considered obesity prevention or lifestyle interventions delivered among Mexican children or adolescents from 1995 onwards in Mexico and were published in English, Spanish or Portuguese. The population included children and adolescents aged zero to 18 years from any ethnicity or gender living in Mexico, including studies involving parents, caregivers or stakeholders if outcomes were measured in children or adolescents. Experimental or quasi experimental study designs in any setting or digital domains were considered for inclusion.	29
Medeiros *et al.* ^(43)^	2022	Up to June 2019	Belgium, Brazil, China, Ecuador, Finland, Greece, Iran, Italy, Netherlands, Norway, Trinidad & Tobago, U.K., U.S.A.	School-based	RCT	Systematic review and meta- analysis	Assess the impact of SBFNE interventions on adolescent food consumption through a systematic review.	Original articles reporting RCTs focusing on SBFNE interventions delivered in school settings, targeting adolescents (aged 10–19 years). The studies must examine the effect of these interventions on adolescent food consumption. There were no restrictions on publication language or time frame.	24 (SR) 11 (MA)
Omidvar *et al.* ^(44)^	2022	Up to 1 October 2021	Australia, Canada, India, Spain, U.K., U.S.A.	School-based	RCT, non-RCT that allocated students individually or in clusters, quasi randomised trials, pre- and post-test, post-test only, case–control	Systematic review	Identify and evaluate interventions promoting children’s Food and Nutrition Literacy (FNLIT) in the school setting, focusing on strategies, implementation methods and effectiveness among primary school children.	Quantitative studies, including case–control studies, pre- and post-interventions, post-test only, randomised and non-randomised controlled trials and quasi randomised trials examining interventions for FNLIT promotion in primary students aged 5–12 years. Studies needed to be available in full-text and in English, featuring interventions targeting FNLIT skills (functional, interactive and critical) for children aged 5–12 years in elementary schools or equivalent educational settings.	19

* Number of primary studies.

To contextualise findings, study characteristics are described below in terms of study design populations and settings and methodological trends.

### Range of study designs used

The included reviews employed diverse study designs (Table 3). Several reviews synthesised randomised controlled trials (RCT) and quasi-experimental studies to assess intervention effectiveness^(14,18,21)^. Others incorporated non-randomised pre-post designs, observational studies, qualitative evaluations or mixed-methods evidence^(12,31,41)^. Early reviews were predominantly narrative in nature, summarising programmatic trends without systematic selection or appraisal criteria^(1–3)^. Over time, systematic review methodologies became common, with meta-analyses emerging in later decades to provide pooled estimates of intervention effects^(17,21,29,30,37,40,43)^.

More recent reviews expanded beyond traditional synthesis approaches, incorporating realist reviews to examine intervention mechanisms of action across contexts and scoping reviews to map emerging domains such as food literacy and digital learning approaches^(36,38)^. This diversity of methodological approaches reflects both the complexity of SBFNE programs and differences in review objectives and outcome measurement strategies.

### Populations and settings covered

The populations examined across reviews varied in age groups, geographic focus and educational settings (Table 3). Some reviews focused primarily on primary school children^(1,21,35,44)^, others examined adolescents^(6,24,39,43)^ and several synthesised evidence across both primary and secondary school populations^(5,23,30,42)^. Most reviews emphasised universal, prevention-oriented SBFNE programs delivered to general student populations and explicitly excluded studies targeting clinical or high-risk subgroups^(9,10,12,22)^.

Geographically, early reviews were concentrated in high-income countries, particularly the United States and Europe^(1,3–5)^. Over time, the scope broadened to include research from low- and middle-income countries and region-specific synthesis, including reviews focused on Latin American^(18,27,28,42)^. While all reviews centred on school-based interventions, several also examined programs that incorporated home^(14,19,36,42)^, community^(19,20,22,33)^ or digital^(41,42)^ components alongside school-based delivery.

### Variation in evidence synthesis approaches across reviews

Across the included reviews, evidence synthesis approaches varied substantially, contributing to heterogeneity in how intervention effectiveness was assessed and reported (Table 3)^(61,62)^. Reviews synthesised a range of primary study designs, including randomised trials, quasi-experimental studies, pre–post evaluations and qualitative investigations. Some reviews emphasised outcome effectiveness, while others focused on implementation processes, contextual factors or theoretical underpinnings.

This methodological diversity underscores the importance of interpreting reported effects in relation to study design, implementation context and review quality. To support this, Table 4 provides a high-level overview of the 1651 primary studies synthesised across reviews, of which 1115 were identified as unique. Several primary studies – such as those by Gortmaker^(63)^, Foster^(64)^, Sahota^(65)^, Perry^(66)^ and Francis^(67)^ – were repeatedly cited across reviews, reflecting their foundational influence within the SBFNE literature.

**Table 4.** Quantitative summary of primary studies included in the forty-four literature reviews Table 4 long description.

	Metric	Value
1	Total literature reviews	44
2	Analytic sample size range	*n* 5 to *n* 268
3	Total primary studies	1651
4	Unique primary studies	1115
5	Duplicate primary studies	536
6	Primary studies with multiple mentions	176
7	Top 5 most frequently cited studies	Count
	Gortmaker, 1999^(63)^	11
	Foster, 2008^(64)^	9
	Sahota, 2001^(65)^	8
	Perry, 1998^(66)^	6
	Francis, 2010^(67)^	6
8	Mean number of studies per review	37·5
9	Median number of studies per review	23·0

### Risk of bias across included reviews

Overall, forty-one reviews (93 %) were classified as high quality^(2–31,33,35–44)^, two (5 %) as medium quality^(32,34)^ and one (2 %) as low quality^(1)^. Across the Snyder quality appraisal phases, reviews demonstrated particularly strong performance in *Design* and *Structuring and Writing* with 99·7 % and 99·1 % of applicable criteria, respectively, scoring as ‘Yes,’ i.e. criterion met. This indicates that most reviews clearly articulated their purpose, methods and reporting structure. In contrast, greater variability was observed in the *Conduct* phase (67 % of criteria scored ‘Yes’), with lower compliance for criteria related to the use of psychosocial theories (18 % ‘Yes’) and theory-based outcomes (36 % ‘Yes’).

Detailed results of the risk of bias assessment are presented in Tables 5 and 6. These assessments informed subsequent synthesis by emphasising findings from higher-quality reviews and supporting sensitivity analyses to assess robustness.

**Table 5.** Quality assessment of included literature reviews (phases 1 and 2) Table 5 long description.

Literature Review Citation			Phase 1: Design							Phase 2: Conduct					
	Year	1	2	3	4	5	6	7	8	9	10	11	12	13	14
Jacobson *et al.* ^(1)^	1959	Y	Y		Y	Y	Y	Y			Y				Y
Whitehead^(2)^	1973	Y	Y	Y	Y	Y	Y	Y			Y	Y	Y	Y	Y
White-Stevens^(3)^	1981	Y	Y	Y	Y	Y	Y	Y			Y	Y	Y	Y	Y
Contento^(4)^	1992	Y	Y	Y	Y	Y	Y	Y	Y	Y	Y	Y	Y	Y	Y
Lytle^(5)^	1995	Y	Y	Y	Y	Y	Y	Y			Y	Y	Y	Y	Y
Hoelscher *et al.* ^(6)^	2002	Y	Y	Y	Y	Y	Y	Y	Y	Y	Y	Y	Y	Y	Y
Woolfe *et al.* ^(7)^	2005	Y	Y	Y	Y	Y	Y	Y			Y	Y	Y	Y	Y
Doak *et al.* ^(8)^	2006	Y	Y	Y	Y	Y	Y	Y			Y	Y	Y	Y	Y
Knai *et al.* ^(9)^	2006	Y	Y	Y	Y	Y	Y	Y			Y	Y	Y	Y	Y
Sharma^(10)^	2006	Y	Y	Y	Y	Y	Y	Y	Y	Y	Y	Y	Y	Y	Y
Peterson & Fox^(11)^	2007	Y	Y	Y	Y	Y	Y	Y			Y	Y	Y	Y	Y
Contento^(12)^	2008	Y	Y	Y	Y	Y	Y	Y			Y	Y	Y	Y	Y
Van Cauwenberghe *et al.* ^(13)^	2010	Y	Y	Y	Y	Y	Y	Y	Y	Y	Y	Y	Y	Y	Y
AlMarzooqi & Nagy^(14)^	2011	Y	Y	Y	Y	Y	Y	Y		Y	Y	Y	Y	Y	Y
Roseman *et al.* ^(15)^	2011	Y	Y	Y	Y	Y	Y	Y		Y	Y	Y	Y	Y	Y
Sharma^(16)^	2011	Y	Y	Y	Y	Y	Y	Y		Y	Y	Y	Y	Y	Y
Evans^(17)^	2012	Y	Y	Y	Y	Y	Y	Y		Y	Y	Y	Y	Y	Y
Verstraeten^(18)^	2012	Y	Y	Y	Y	Y	Y	Y		Y	Y	Y	Y	Y	Y
Hackman *et al.* ^(19)^	2014	Y	Y	Y	Y	Y	Y	Y	Y	Y	Y	Y	Y	Y	Y
Rush *et al.* ^(20)^	2014	Y	Y	Y	Y	Y	Y	Y			Y	Y	Y	Y	Y
Dudley^(21)^	2015	Y	Y	Y	Y	Y	Y	Y			Y	Y	Y	Y	Y
Shirley *et al.* ^(22)^	2015	Y	Y	Y	Y	Y	Y	Y			Y	Y	Y	Y	Y
Yip *et al.* ^(23)^	2015	Y	Y	Y	Y	Y	Y	Y			Y	Y	Y	Y	Y
Meiklejohn *et al.* ^(24)^	2016	Y	Y	Y	Y	Y	Y	Y			Y	Y	Y	Y	Y
Murimi *et al.* ^(25)^	2018	Y	Y	Y	Y	Y	Y	Y			Y	Y	Y	Y	Y
Muzaffar *et al.* ^(26)^	2018	Y	Y	Y	Y	Y	Y	Y			Y	Y	Y	Y	Y
Chaudhary *et al.* ^(27)^	2020	Y	Y	Y	Y	Y	Y	Y			Y	Y	Y	Y	Y
Chavez & Nam^(28)^	2020	Y	Y	Y	Y	Y	Y	Y			Y	Y	Y	Y	Y
Cotton *et al.* ^(29)^	2020	Y	Y	Y	Y	Y	Y	Y			Y	Y	Y	Y	Y
Dabravolskaj *et al.* ^(30)^	2020	Y	Y	Y	Y	Y	Y	Y	Y	Y	Y	Y	Y	Y	Y
Gonzalez-Jaramillo *et al.* ^(31)^	2020	Y	Y	Y	Y	Y	Y	Y		Y	Y	Y	Y		Y
Roseman *et al.* ^(32)^	2020	Y	Y	Y	Y	Y	Y	Y			Y	Y	Y		Y
Shapu *et al.* ^(33)^	2020	Y	Y	Y	Y	Y	Y	Y			Y	Y	Y	Y	Y
Bennett *et al.* ^(34)^	2021	Y	Y	Y	Y	Y	Y	Y			Y	Y	Y		Y
Charlton *et al.* ^(35)^	2021	Y	Y	Y	Y	Y	Y	Y			Y	Y	Y	Y	Y
Ijaz *et al.* ^(36)^	2021	Y	Y	Y	Y	Y	Y	Y			Y	Y	Y	Y	Y
Ismail *et al.* ^(37)^	2021	Y	Y	Y	Y	Y	Y	Y			Y	Y	Y	Y	Y
Kelly & Nash^(38)^	2021	Y	Y	Y	Y	Y	Y	Y	Y	Y	Y	Y	Y	Y	Y
Rose *et al.* ^(39)^	2021	Y	Y	Y	Y	Y	Y	Y		Y	Y	Y	Y	Y	Y
Singhal *et al.* ^(40)^	2021	Y	Y	Y	Y	Y	Y	Y			Y	Y	Y	Y	Y
Tallon *et al.* ^(41)^	2021	Y	Y	Y	Y	Y	Y	Y			Y	Y	Y	Y	Y
Aceves- Martins *et al.* ^(42)^	2022	Y	Y	Y	Y	Y	Y	Y		Y	Y	Y	Y	Y	Y
Medeiros *et al.* ^(43)^	2022	Y	Y	Y	Y	Y	Y	Y	Y		Y	Y	Y	Y	Y
Omidvar *et al.* ^(44)^	2022	Y	Y	Y	Y	Y	Y	Y		Y	Y	Y	Y	Y	Y
**Total = ‘Yes’**		44, 100 %	44, 100 %	43, 98 %	44, 100 %	44, 100 %	44, 100 %	44, 100 %	8, 18 %	16, 36 %	44, 100 %	43, 98 %	43, 98 %	40, 91 %	44, 100 %
**Total = ‘No’**		0	0	1	0	0	0	0	36	28	0	1	1	4	0
Phase 1: Design 1. Contribution to the field 2. Clarify of motivation, purpose and question 3. Account for previous reviews and literature 4. Clarity of approach/methodology 5. Appropriateness of approach 6. Description of motivation of methodology and search strategy 7. Types of educational interventions					Phase 2: Conduct 8. Use of psychosocial theories in development of intervention 9. Criteria for measuring theory-based determinants and behaviours as outcomes 10. Appropriateness of search process 11. Accuracy of search process description 12. Transparency in inclusion and exclusion 13. Measures for research quality 14. Trustworthiness of final sample										

**Table 6.** Quality assessment of included literature reviews (phases 3 & 4) Table 6 long description.

Literature review citation		Phase 3: Data abstraction					Phase 4: Structuring and writing the review					Totals/ review Phases 1–4	
	Year	15	16	17	18	19	20	21	22	23	24	Yes	No
Jacobson *et al.* ^(1)^	1959					Y	Y		Y	Y	Y	**13**	**11**
Whitehead^(2)^	1973	Y	Y	Y	Y	Y	Y	Y	Y	Y	Y	**22**	**2**
White-Stevens^(3)^	1981	Y	Y	Y	Y	Y	Y	Y	Y	Y	Y	**22**	**2**
Contento^(4)^	1992	Y	Y	Y	Y	Y	Y	Y	Y	Y	Y	**24**	**0**
Lytle^(5)^	1995	Y	Y	Y	Y	Y	Y	Y	Y	Y	Y	**22**	**2**
Hoelscher *et al.* ^(6)^	2002	Y	Y	Y	Y	Y	Y	Y	Y	Y	Y	**24**	**0**
Woolfe *et al.* ^(7)^	2005	Y	Y	Y	Y	Y	Y	Y	Y	Y	Y	**22**	**2**
Doak *et al.* ^(8)^	2006	Y	Y	Y	Y	Y	Y	Y	Y	Y	Y	**22**	**2**
Knai *et al.* ^(9)^	2006	Y	Y	Y	Y	Y	Y	Y	Y	Y	Y	**22**	**2**
Sharma^(10)^	2006	Y	Y	Y	Y	Y	Y	Y	Y	Y	Y	**24**	**0**
Peterson & Fox^(11)^	2007	Y	Y	Y	Y	Y	Y	Y	Y	Y	Y	**22**	**2**
Contento^(12)^	2008	Y	Y	Y	Y	Y	Y	Y	Y	Y	Y	**22**	**2**
Van Cauwenberghe *et al.* ^(13)^	2010	Y	Y	Y	Y	Y	Y	Y	Y	Y	Y	**24**	**0**
AlMarzooqi & Nagy^(14)^	2011	Y	Y		Y	Y	Y	Y	Y	Y	Y	**22**	**2**
Roseman *et al.* ^(15)^	2011	Y	Y	Y	Y	Y	Y	Y	Y	Y	Y	**23**	**1**
Sharma^(16)^	2011	Y	Y	Y	Y	Y	Y	Y	Y	Y	Y	**23**	**1**
Evans^(17)^	2012	Y	Y	Y	Y	Y	Y	Y	Y	Y	Y	**23**	**1**
Verstraeten^(18)^	2012	Y	Y	Y	Y	Y	Y	Y	Y	Y	Y	**23**	**1**
Hackman *et al.* ^(19)^	2014	Y	Y	Y	Y	Y	Y	Y	Y	Y	Y	**24**	**0**
Rush *et al.* ^(20)^	2014	Y	Y		Y	Y	Y	Y	Y	Y	Y	**21**	**3**
Dudley^(21)^	2015	Y	Y	Y	Y	Y	Y	Y	Y	Y	Y	**22**	**2**
Shirley *et al.* ^(22)^	2015	Y	Y	Y	Y	Y	Y	Y	Y	Y	Y	**22**	**2**
Yip *et al.* ^(23)^	2015	Y	Y	Y	Y	Y	Y	Y	Y	Y	Y	**22**	**2**
Meiklejohn *et al.* ^(24)^	2016	Y	Y	Y	Y	Y	Y	Y	Y	Y	Y	**22**	**2**
Murimi *et al.* ^(25)^	2018	Y	Y	Y	Y	Y	Y	Y	Y	Y	Y	**22**	**2**
Muzaffar *et al.* ^(26)^	2018	Y	Y	Y	Y	Y	Y	Y	Y	Y	Y	**22**	**2**
Chaudhary *et al.* ^(27)^	2020	Y	Y	Y	Y	Y	Y	Y	Y	Y	Y	**22**	**2**
Chavez & Nam^(28)^	2020	Y	Y	Y	Y	Y	Y	Y	Y	Y	Y	**22**	**2**
Cotton *et al.* ^(29)^	2020	Y	Y	Y	Y	Y	Y	Y	Y	Y	Y	**22**	**2**
Dabravolskaj *et al.* ^(30)^	2020	Y	Y	Y	Y	Y	Y	Y	Y	Y	Y	**24**	**0**
Gonzalez-Jaramillo *et al.* ^(31)^	2020	Y	Y		Y	Y	Y	Y	Y	Y	Y	**21**	**3**
Roseman *et al.* ^(32)^	2020	Y	Y		Y	Y	Y	Y	Y	Y	Y	**20**	**4**
Shapu *et al.* ^(33)^	2020	Y	Y	Y	Y	Y	Y	Y	Y	Y	Y	**22**	**2**
Bennett *et al.* ^(34)^	2021	Y	Y		Y	Y	Y	Y	Y	Y	Y	**20**	**4**
Charlton *et al.* ^(35)^	2021	Y	Y	Y	Y	Y	Y	Y	Y	Y		**21**	**3**
Ijaz *et al.* ^(36)^	2021	Y	Y	Y	Y	Y	Y	Y	Y	Y	Y	**22**	**2**
Ismail *et al.* ^(37)^	2021	Y	Y	Y	Y	Y	Y	Y	Y	Y	Y	**22**	**2**
Kelly & Nash^(38)^	2021	Y	Y	Y	Y	Y	Y	Y	Y	Y	Y	**24**	**0**
Rose *et al.* ^(39)^	2021	Y	Y	Y	Y	Y	Y	Y	Y	Y	Y	**23**	**1**
Singhal *et al.* ^(40)^	2021	Y	Y	Y	Y	Y	Y	Y	Y	Y	Y	**22**	**2**
Tallon *et al.* ^(41)^	2021	Y	Y	Y	Y	Y	Y	Y	Y	Y	Y	**22**	**2**
Aceves- Martins *et al.* ^(42)^	2022	Y	Y	Y	Y	Y	Y	Y	Y	Y	Y	**23**	**1**
Medeiros *et al.* ^(43)^	2022	Y	Y	Y	Y	Y	Y	Y	Y	Y	Y	**23**	**1**
Omidvar *et al.* ^(44)^	2022	Y	Y	Y	Y	Y	Y	Y	Y	Y	Y	**23**	**1**
**Total = ‘Yes’**		43, 98 %	43, 98 %	38, 86 %	43, 98 %	44, 100 %	44, 100 %	43, 98 %	44, 100 %	44, 100 %	43, 98 %		
**Total = ‘No’**		1	1	6	1	0	0	1	0	0	1		
Phase 3: Data abstraction 15. Appropriateness of Data Abstracted 16. Accuracy of Data Abstraction Process 17. Measures of Quality Data Abstraction 18. Appropriateness of Data Analysis Technique 19. Transparency in Analysis Process			Phase 4: Structuring and writing the review 20. Coherence in Organization 21. Description of Review Method and Replicability 22. Clarity in Reporting Results 23. Synthesis and Contribution 24. Inclusion of Further Research Questions and Usability Results										

### Identification and definition of key educational and programmatic components

Across the included literature reviews, synthesis of reported intervention characteristics enabled the identification and consolidation of twenty distinct educational and programmatic components associated with SBFNE effectiveness. Components were defined as core educational or programmatic features considered essential to program design, implementation, sustainability or evaluation and were identified based on repeated inclusion across reviews and explicit linkage to dietary behaviour outcomes.

The twenty identified components are: *Family Engagement* (identified in forty-two of forty-four reviews); *Focused on Behaviour Change* (identified in forty-two reviews); *Multicomponent Approaches* (identified in forty-two reviews); *Curriculum Integration* (identified in forty reviews); *Healthful Changes in School Food & Physical Activity Environments* (identified in forty reviews); *Sequential and Sufficient Duration and Intensity [Adequate Dose]* (identified in forty reviews); *Evaluations: Quantitative and Qualitative, Before, During and After Intervention* (identified in thirty-eight reviews); *Experiential Learning Activities* (identified in thirty-seven reviews); *Connections to School Meal*s (identified in thirty-two reviews); *Theory- and Evidenced Based* (identified in thirty-two reviews); *Age-Appropriate & Context-Specific Learning Activities* (identified in thirty-one reviews); *Professional Development* (identified in thirty reviews); *Community Engagement* (identified in twenty-eight reviews); *Administrative Support* (identified in twenty-four reviews); *Student Goal setting, Self-Regulation & Self-Assessment* (identified in twenty-four reviews); *Multimedia Technology Engagement* (identified in twenty reviews); *Culturally Inclusive Practices* (identified in nineteen reviews); *Peer Support* (identified in eighteen reviews); *Intervention Tailored to Students’ Economic Status* (identified in fourteen reviews); and *Advocates for Environmental Sustainability* (identified in three reviews).

The full set of identified components and their standardised operational definitions are presented in Table 7. Definitions were refined to ensure consistency across reviews that varied widely in terminology, scope and methodological approach, allowing components to be systematically compared and synthesised across diverse intervention contexts.

**Table 7.** Names and definitions of the twenty key educational and programmatic components that make SBFNE effective Table 7 long description.

#	Component name	Definition
1	Culturally inclusive practices	Adapting food and nutrition education to reflect and respect the cultural identities, traditions, and experiences of diverse communities, making learning more relevant, inclusive, and effective while fostering appreciation of both one’s own and other cultures.
2	Intervention tailored to students’ economic status	Adjusting educational content, activities or recommended behaviours to reflect students’ economic circumstances, including food affordability, access and resource constraints. Across reviews, this component was operationalised through strategies such as emphasising low-cost food options, aligning lessons with foods commonly available through school meals or food assistance programs and avoiding recommendations that assume discretionary income or specialised food access.
3	Advocates for environmental sustainability	Educational content or programmatic approaches that explicitly link food choices to environmental outcomes, including lessons on food systems, sustainability or food justice. Across reviews, this component was operationalised through curriculum elements that addressed the environmental impacts of food production, consumption or waste alongside nutrition education.
4	Focused on behaviour change	Interventions that explicitly target-specific dietary behaviours or practices, rather than general nutrition awareness or information provision. Across reviews, this component was operationalised through strategies that emphasised skill-building, goal setting, self-monitoring or practice-oriented activities designed to support observable dietary behaviour change.
5	Multicomponent approaches	Interventions that combine classroom-based food and nutrition education with one or more additional components – such as *Experiential Learning Activities* (e.g. cooking or gardening), *Healthful Changes in School Food & Physical Activity Environment,* or *Family Engagement* – implemented in a coordinated manner to reinforce dietary behaviour change.
6	Healthful changes in school food and physical activity environments	Initiatives that modify the school environment to support healthier behaviours, including changes to food service, cafeterias, vending machines, school stores or food marketing, as well as adjustments to physical activity spaces or opportunities that facilitate healthier dietary and activity-related choices.
7	Family engagement	Involving parents or caregivers as part of the intervention through strategies such as take-home materials, family-oriented activities, participation in school-based events or engagement in curriculum-linked activities that extend nutrition education beyond the classroom.
8	Student goal setting, self-regulation and self-assessment	Intervention components that engage students in setting dietary goals and monitoring their own behaviours through tools such as food diaries, questionnaires, checklists or reflective activities linked to program objectives.
9	Evaluations: quantitative and qualitative, before, during and after	Program evaluation approaches that include quantitative and qualitative data collection at multiple time points (pre-, during and post-intervention) and assess multiple domains, including student-level outcomes (e.g. dietary behaviours, knowledge or attitudes), program implementation and fidelity, facilitator or educator delivery and contextual factors such as feasibility, acceptability and sustainability.
10	Community engagement	The involvement of community-based partners – such as local organisations, food producers, health professionals or community food initiatives – in the design, delivery or reinforcement of school-based food and nutrition education. These partnerships extend learning beyond the classroom and support alignment between school-based education and the broader food environment in which students and families live.
11	Multimedia technology engagement	The use of digital or multimedia tools – such as educational games, websites, videos or other technology-based platforms – to support the delivery or reinforcement of school-based food and nutrition education.
12	Sequential and sufficient duration and intensity (adequate dose)	The structuring of interventions to occur in a logical sequence and over a duration and intensity considered sufficient to reinforce learning and support behaviour change, rather than brief or standalone exposures.
13	Theory- and evidenced based	The grounding of food and nutrition education interventions in established behavioural theories, learning models and empirical evidence, which informs the selection and organization of strategies intended to influence dietary behaviours.
14	Age-appropriate & context-specific learning activities	Delivering food and nutrition education through learning activities tailored to the developmental stage of students, while also considering contextual factors such as geographical location, family dynamics, personal experiences with food, health conditions and media influence. This approach ensures content is not only age aligned but also resonates with the diverse experiences and challenges students may encounter, enhancing engagement and effectiveness.
15	Experiential learning activities	Employing a hands-on approach to education with learning activities that actively engage students in action, experience, reflection and critical thinking. This cyclical process of acting ↔ experiencing ↔ reflecting ↔ thinking allows students to deeply connect with and internalise food and nutrition concepts, fostering a more profound understanding and application of the knowledge gained.
16	Peer support	The incorporation of peer-based strategies within food and nutrition education, including peer discussion, collaborative activities or peer-led elements, that leverage social interaction and shared learning among students.
17	Administrative support	Support from school leadership and relevant institutional stakeholders – such as principals, school administrators, district offices or education authorities – that facilitates the planning, implementation and maintenance of food and nutrition education programs. This support may include policy alignment, resource allocation, scheduling flexibility or formal endorsement of program activities.
18	Curriculum integration	The integration of food and nutrition education within the existing school curriculum, either as a standalone subject or embedded across academic areas (e.g. science, mathematics or humanities). This approach reflects alignment with curricular standards and schedules, supporting consistent delivery of food and nutrition content within routine instructional time.
19	Professional development	Training and structured learning opportunities provided to educators and program staff to build knowledge, skills and confidence related to food and nutrition education. Professional development may include instruction, guided reflection or facilitated program review and may be delivered by food and nutrition professionals (e.g. registered dietitian nutritionists) or other qualified trainers. These activities are intended to support high-quality implementation, evaluation and continuous improvement of school-based food and nutrition education programs, whether delivered by classroom teachers or external partners.
20	Connections to school meals	The intentional alignment between school-based food and nutrition education and the school meals program, such that foods served, messages conveyed and practices reinforced during meals are consistent with educational content. This alignment supports experiential reinforcement of nutrition concepts, increases exposure to healthier foods and strengthens the likelihood that nutrition education messages are translated into students’ food choices within the school setting.

*Note:* Components are listed in the order in which they were identified during synthesis and are numbered for reference only; numbering does not indicate relative importance, prevalence or strength of evidence.

### Quantitative synthesis of component prevalence and reported effects

A quantitative synthesis was conducted to examine the prevalence of each component across the included reviews and the reported direction of associated dietary outcomes. Table 8 presents a matrix indicating whether each component was addressed within a given review and the direction of its reported effect on dietary behaviour. Symbols denote effect classification, where ‘**+**’ indicates a net positive effect, ‘O’ indicates no effect or an inconclusive effect, ‘−’ indicates a net negative effect and a blank cell indicates that the component was not analysed within that review.

**Table 8.** Quantitative synthesis of component prevalence and reported effects Table 8 long description.

		Prevalence and reported impact of each component									
Chronological literature review citation		1	2	3	4	5	6	7	8	9	10
1959	Jacobson *et al.* ^(1)^	+	+		+	+	+	+	+	+	+
1973	Whitehead^(2)^	○	+		+	+	+	+	+	+	+
1981	White-Stevens^(3)^	○	○		○	○	○	○	○	○	○
1992	Contento^(4)^		+		+	+	+	+/-	+	+	
1995	Lytle^(5)^	○			+		+	+	+		+
2002	Hoelscher *et al.* ^(6)^	○			+	+	+	+	+	+	+
2005	Woolfe *et al.* ^(7)^	+	+		+	+	+	+	+	+	+
2006	Doak *et al.* ^(8)^	+	+		+	+	+	+		+	+
2006	Knai *et al.* ^(9)^				+	+	+	+	○	+	+
2006	Sharma^(10)^		+		+	+	+	+		+	
2007	Peterson & Fox^(11)^		+		+	+	+	+		+	+
2008	Contento^(12)^	○		+	+	+	+	+	+	+	+
2010	Van Cauwenberghe *et al.* (2010)^(13)^	+/○	+/○		+	+/○	+	○		+	
2011	AlMarzooqi & Nagy^(14)^				+	+	+	+	○	+	+
2011	Roseman *et al.* ^(15)^	+			+	+	+	+/-	+	+	+
2011	Sharma^(16)^				+	+	+	+	+	+	+
2012	Evans^(17)^				+	+	+	+	○	+	○
2012	Verstraeten^(18)^				+	+	+	+		+	+
2014	Hackman *et al.* ^(19)^	+			+	+	+	○	○	+	+
2014	Rush *et al.* ^(20)^	+			+	+	+	+		+	+
2015	Dudley^(21)^				+	+		+/-	+	+	
2015	Shirley *et al.* ^(22)^				+	+	+	+		+	+
2015	Yip *et al.* ^(23)^	+			+	+	+				
2016	Meiklejohn *et al.* ^(24)^				+	+	+	+	+		+
2018	Murimi *et al.* ^(25)^				+	+	+	+		+	
2018	Muzaffar *et al.* ^(26)^				+	+		+		○	○
2020	Chaudhary *et al.* ^(27)^	+	+		+	+	+	+		+	
2020	Chavez & Nam^(28)^				+	+	+	+		+	
2020	Cotton *et al.* ^(29)^				+	+	+	+			
2020	Dabravolskaj *et al.* ^(30)^						+		+		○
2020	Gonzalez-Jaramillo *et al.* ^(31)^				+	+	+	+	+	+	+
2020	Roseman *et al.* ^(32)^	+			+	+	+	+	+	+	+
2020	Shapu *et al.* ^(33)^		+		+	+		+		+	+
2021	Bennett *et al.* ^(34)^	○		+	+	+	+	○		○	+
2021	Charlton *et al.* ^(35)^				+	+	+	+	+	+	+
2021	Ijaz *et al.* ^(36)^	+/○	+		+	+	+/○	+	+	+	
2021	Ismail *et al.* ^(37)^		+		+	+	+	+		+	
2021	Kelly & Nash^(38)^				+	+		+	+	+	+
2021	Rose *et al.* ^(39)^		+		+	+	+	+	+	+	
2021	Singhal *et al.* ^(40)^				+	+	+	+		+	+
2021	Tallon *et al.* ^(41)^				+	+	+	+	+	+	
2022	Aceves- Martins *et al.* ^(42)^	○			+	+	+	+		+	
2022	Medeiros *et al.* ^(43)^				+	+	+	+		+	
2022	Omidvar *et al.* ^(44)^	+		+		+	○	+			
Reviews analysing component #, %		19, 43 %	14, 32 %	3, 7 %	42, 95 %	42, 95 %	40, 91 %	42, 95 %	24, 55 %	38, 86 %	28, 64 %
Positive effect #, %		10, 53 %	12, 86 %	3, 100 %	41, 98 %	40, 95 %	37, 92 %	36, 86 %	19, 79 %	35, 92 %	24, 86 %
Mixed effect #, %		2, 11 %	1, 7 %			1, 2 %	1, 3 %	3, 7 %			
No or inconclusive effect #, %		7, 37 %	1, 7 %		1, 2 %	1, 2 %	2, 5 %	3, 7 %	5, 21 %	3, 8 %	4, 14 %

		Prevalence and reported impact of each component										Included	
Chronological literature review citation		11	12	13	14	15	16	17	18	19	20	#	%
1959	Jacobson *et al.* ^(1)^				+	+	+	+	+	+	+	16	80 %
1973	Whitehead^(2)^		+		+	+		+	+	+	+	16	80 %
1981	White-Stevens^(3)^	○			○	○	○	○	○		○	16	80 %
1992	Contento^(4)^		+	+	+	+	+		+	+/○	+	15	75 %
1995	Lytle^(5)^	+	+	+	+	+	+	+		+/-	+	15	75 %
2002	Hoelscher *et al.* ^(6)^	○	+	+	+	+	+	+	+	+	+	18	90 %
2005	Woolfe *et al.* ^(7)^	+	+	+		+		+	+	○		16	80 %
2006	Doak *et al.* ^(8)^		+	+	+	+		+	+			14	70 %
2006	Knai *et al.* ^(9)^		+	+		+	○	+	+	+	+	15	75 %
2006	Sharma^(10)^		+	+	+	+		+	+	+	+	14	70 %
2007	Peterson & Fox^(11)^	+	+	+		+		+	+	+	+	15	75 %
2008	Contento^(12)^	○	+	+	+	+	+	+	–	+	+	19	95 %
2010	Van Cauwenberghe *et al.* (2010)^(13)^		○		+		+				+	11	55 %
2011	AlMarzooqi & Nagy^(14)^		○	+/○				+	○	○		12	60 %
2011	Roseman *et al.* ^(15)^	+	+		+	○			+	○		14	65 %
2011	Sharma^(16)^		+	○	+	+		+	+	+	+	15	75 %
2012	Evans^(17)^		+	+		+		+	+	+	+	14	70 %
2012	Verstraeten^(18)^		+	+	+	+		+	+	+	+	14	70 %
2014	Hackman *et al.* ^(19)^	○	+	+	+	+		+	+	+	+	17	85 %
2014	Rush *et al.* ^(20)^	+	+	+	+	+	+	+	+	+	+	17	85 %
2015	Dudley^(21)^	+	○	○	+	+			+			11	55 %
2015	Shirley *et al.* ^(22)^		+		○				+	○	+	11	55 %
2015	Yip *et al.* ^(23)^		+	○		○	+		○			9	45 %
2016	Meiklejohn *et al.* ^(24)^	+	+	+			+	○	+		+	13	65 %
2018	Murimi *et al.* ^(25)^	+	+	+	+	+			+	+	+	13	65 %
2018	Muzaffar *et al.* ^(26)^		+	○	+	+			+		+	11	55 %
2020	Chaudhary *et al.* ^(27)^		+		+	+	+		+	+	+	14	70 %
2020	Chavez & Nam^(28)^		+/-	+	+/○	+			+	○		11	55 %
2020	Cotton *et al.* ^(29)^		+	+		+		+	+	+	+	11	55 %
2020	Dabravolskaj *et al.* ^(30)^		+			+		+	+		+	8	40 %
2020	Gonzalez-Jaramillo *et al.* ^(31)^	+	+	○		+	+	+	+	+	+	16	80 %
2020	Roseman *et al.* ^(32)^	+	+		○		+		+	+	○	15	75 %
2020	Shapu *et al.* ^(33)^		○	+	○	+						10	50 %
2021	Bennett *et al.* ^(34)^		○	+	+	+			+	+	+	15	75 %
2021	Charlton *et al.* ^(35)^		+	+	+	+		+	+	+	+	15	75 %
2021	Ijaz *et al.* ^(36)^	○	+		+	+	+		+	+		15	75 %
2021	Ismail *et al.* ^(37)^		+	+			+	○	+	○	○	13	65 %
2021	Kelly & Nash^(38)^	+			+	+			+		+	11	55 %
2021	Rose *et al.* ^(39)^	+	○	+	+	+	+		+		+	15	75 %
2021	Singhal *et al.* ^(40)^		+		○	○			+		+	11	55 %
2021	Tallon *et al.* ^(41)^	+	+	+	+				+			11	55 %
2022	Aceves- Martins *et al.* ^(42)^	○	+	+		○	+				+	12	60 %
2022	Medeiros *et al.* ^(43)^		+	+	+	+		+	+	+		12	60 %
2022	Omidvar *et al.* ^(44)^	+		+		+			+	+		10	50 %
		20, 45 %	40, 91 %	32, 73 %	31, 70 %	37, 84 %	18, 41 %	24, 55 %	40, 91 %	30, 68 %	32, 73 %		
		14, 70 %	33, 82 %	26, 81 %	25, 81 %	32, 86 %	16, 89 %	21, 88 %	36, 90 %	22, 73 %	29, 91 %		
			1, 3 %	1, 3 %	1, 3 %				1, 2 %	2, 7 %			
		6, 30 %	6, 15 %	5, 16 %	5, 16 %	5¸14 %	2, 11 %	3, 12 %	3, 8 %	6, 20 %	3, 9 %		

Component identification number and corresponding name				Component effectiveness rating scale:
Component 1:	Culturally inclusive	Component 11:	Multimedia technology engagement	+ = net positive effect - = net negative effect ○ = no effect/inconclusive +/○ = positive and no effect/inconclusive +/- = positive and negative effect Blank = component was not analysed
Component 2:	Intervention tailored to students’ economic status	Component 12:	Sequential and sufficient duration and intensity	
Component 3:	Advocates for environmental sustainability	Component 13:	Theory- and evidenced based	
Component 4:	Focused on behaviour change	Component 14:	Age-appropriate learning activities	
Component 5:	Multicomponent	Component 15:	Experiential learning activities	
Component 6:	Healthful changes in school food and physical activity environments	Component 16:	Peer support	
Component 7:	Family engagement	Component 17:	Administrative support	
Component 8:	Student goal setting, self-regulation and self-assessment	Component 18:	Curriculum integration	
Component 9:	Evaluations: quantitative and qualitative, before, during and after	Component 19:	Professional development	
Component 10:	Community engagement	Component 20:	Connections to school meals	

**Figure 2.**
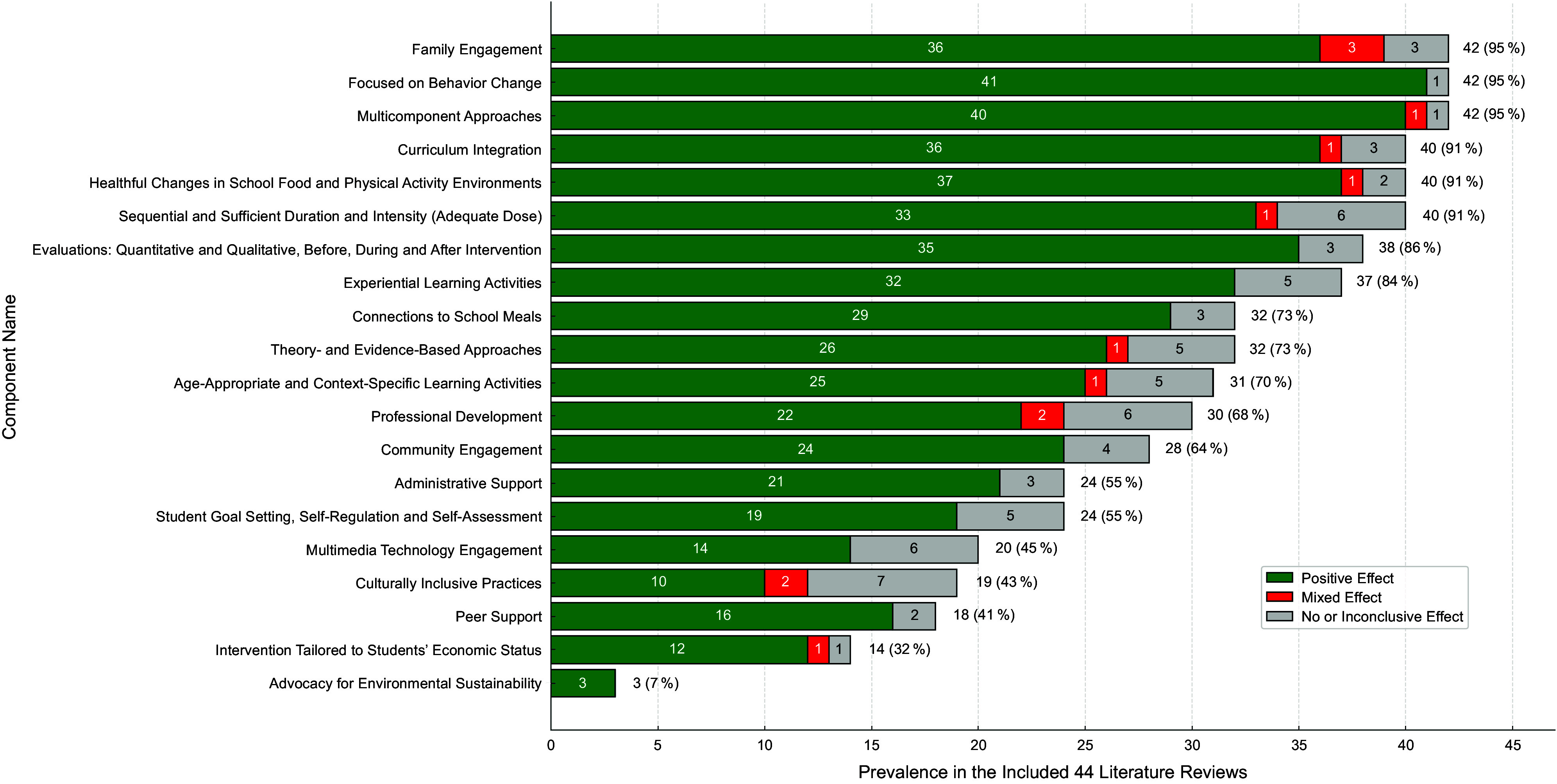
Frequency of components discussed in literature reviews.

Components such as *Focused on Behaviour Change*, *Multicomponent Approaches*, *Family Engagement* and *Integration into the School Curriculum* were among the most frequently identified across reviews and were consistently associated with positive dietary behaviour change. In contrast, components including *Advocates for Environmental Sustainability* and I*nterventions Tailored to Students’ Economic Status* appeared less frequently, although they demonstrated positive effects in the reviews in which they were examined. Several components, including *Culturally Inclusive Practices* and *Professional Development*, showed mixed or inconclusive findings, often reflecting variability in implementation fidelity, programme duration or outcome measurement across studies. Figure 2 illustrates the distribution of the twenty identified SBFNE components across included reviews, highlighting differences in the extent to which components were addressed.

Among the components most frequently identified across reviews, *Multicomponent Approaches* and *Family Engagement* are expanded on here because reviews consistently described substantial variability in how these components were operationalised, warranting brief narrative clarification beyond tabular definitions.

#### Multicomponent approaches

Across included reviews, ‘multicomponent’ referred to programs that combined SBFNE with at least one additional component – most commonly *Experiential Learning Activity* (e.g., cooking or gardening), *Healthful Changes in School Food & Physical Activity Environments* and/or *Family Engagement*. Across the evidence, such combinations were more consistently associated with improvements in dietary behaviours than education delivered in isolation, likely because skills and messages introduced in lessons were reinforced through opportunities to practice, observe and access healthier options within the school context. Where reviews commented on variation, *Multicomponent Approaches* appeared most effective when components were intentionally aligned rather than simply co-occurring, such that educational content, environmental supports and reinforcement activities targeted the same behavioural goals.

#### Family engagement

*Family Engagement* was described across reviews as a spectrum, ranging from low-intensity strategies (e.g. newsletters or take-home materials) to more active approaches (e.g. interactive home activities, caregiver participation in school-based events or structured parent sessions). Reviews generally indicated that more participatory forms of family engagement were more consistently associated with dietary behaviour change than passive information-sharing alone, while also acknowledging challenges related to feasibility and reach. Several reviews further noted that the influence of *Family Engagement* varied by student age, with stronger and more consistent effects reported in interventions targeting younger children compared with adolescents, underscoring the importance of aligning family strategies with developmental stage and implementation constraints.

Other highly prevalent components – such as *Focused on Behaviour Change*, *Curriculum Integration*, *Healthful Changes in School Food and Physical Activity Environments* and *Adequate Program Dose* – were more consistently defined across reviews and are therefore fully detailed in Tables 7, 8 and 9.

## Discussion

### Interpretation of findings

This umbrella review identified twenty key educational and programmatic components that contribute to effective school-based food and nutrition education (SBFNE) programs^(1–44)^. Components most consistently linked to positive dietary behaviour change – such as *Family Engagement, Focused on Behaviour Change, Multicomponent Approaches and Curriculum Integration* – were highly prevalent across reviews and align with prior evidence emphasising comprehensive, behaviour-focused strategies in health education^(21,40,43)^.

Other components, including *Environmental Sustainability* and *Cultural Inclusivity*, were examined less frequently across reviews, but were consistently associated with positive dietary outcomes in the studies that assessed them^(12,34,44)^. These components have been more explicitly examined in more recent SBFNE literature, which may partially explain their lower representation across the full review sample. Although the evidence base for these components is smaller than for more established approaches, the findings to date suggest they can support dietary behaviour change in contextually relevant ways when intentionally integrated into programmes^(45)^.

Importantly, no component was consistently linked to negative outcomes. However, some exhibited inconclusive or variable effectiveness, particularly when implementation fidelity varied or when programme durations were too short to observe sustained changes^(19,26,34)^. *Multimedia Technology Engagement* yielded mixed results across multiple reviews, likely due to variability in implementation quality, differences in student access to digital resources or inconsistencies in how multimedia tools were integrated into instruction^(36,42)^. This variability underscores the need to consider the context: programmes using technology or culturally tailored approaches must ensure appropriate implementation for their specific student populations.

### Outcome-specific effects

Findings also indicate that some components were more strongly associated with particular dietary outcomes. *Experiential Learning Activities* and *Family Engagement* were frequently linked to increased fruit and vegetable consumption, while *Healthful Changes in School Food and Physical Activity Environments* (policy changes) were more associated with reducing highly processed food intake^(17,24,32)^. However, a subset of components – including *Multicomponent Approaches* and *Focused on Behaviour Change* – demonstrated positive effects across both dietary outcomes, suggesting their broad applicability^(12,25)^.

### Strengths

This umbrella review has several notable strengths. First, it synthesises evidence across diverse SBFNE programs, populations and methodological approaches. Second, the use of PRISMA 2020 guidelines and a structured risk-of-bias assessment enhances methodological rigor and transparency. By synthesising evidence at the level of educational and programmatic components rather than individual interventions, this review provides insight into transferable features of effective SBFNE programs. Despite variation in study design and geographic context, the consistency with which core components emerged across reviews strengthens confidence in their relevance. Finally, the findings are relevant to educators, program developers and policymakers, offering practical insights to inform program design and decision-making.

### Limitations

Several limitations should be considered when interpreting the findings of this umbrella review, many of which are inherent to synthesising evidence across existing reviews rather than primary studies^(53,57,68)^. Umbrella reviews aggregate findings across reviews that vary widely in scope, inclusion criteria, study design and analytic approach, which introduces challenges in comparability and interpretation. Substantial heterogeneity existed across the included reviews with respect to intervention content, intensity, duration, target populations and outcome measurement, limiting the ability to draw uniform conclusions across programs.

Overlap of primary studies across reviews represents an additional challenge as frequently cited interventions may be disproportionately represented in the synthesis, potentially inflating the apparent strength of evidence for certain components^(68)^. Reliance on peer-reviewed literature may also contribute to publication bias, underrepresenting community-based or practice-driven interventions that are less likely to be formally published^(55,56)^. In addition, most reviews focused on high-income settings, and few disaggregated findings by socio-demographic characteristics, constraining conclusions about equity and generalisability.

Interpretation is further complicated by variability in implementation fidelity and contextual factors across programmes. For example, there were differences in how dietary outcomes were measured and how programme components were defined and operationalised across reviews – including the use of varied terminology to describe unhealthy or highly processed foods. For programmes targeting adolescents, variability in developmental stage, autonomy and school environments may have strongly influenced engagement and outcomes. Together, these factors underscore the need for cautious interpretation of effect consistency across reviews and highlight the importance of context when translating findings into practice.

### Implications for practice, policy and future research

#### Practice and policy

Findings from this umbrella review reinforce the importance of embedding the components *Family Engagement, Focused on Behaviour Change* and *Multicomponent Approaches* into school wellness policies and nutrition education initiatives^(70,71)^. These components consistently emerged across reviews as central to effective dietary behaviour change and are well suited for integration into existing school health frameworks. Policymakers should consider incorporating these elements into national and regional food and nutrition education standards to support programme sustainability and scalability^(72–75)^.

The underrepresentation of diverse student populations across the reviewed literature further highlights an important limitation of the existing evidence base. Schools and education systems should explicitly address socio-economic context, cultural relevance and neurodiversity when designing and implementing programmes, ensuring that identified components are applied in ways that promote equitable access, engagement and effectiveness across diverse learning environments.

#### Future research

Several priorities for future research emerge from this component-level synthesis. First, there is a need to expand research evaluating SBFNE components in low- and middle-income countries, where food environments, educational infrastructure and implementation constraints may differ substantially from those in high-income settings. Broadening the geographic and socio-economic scope of future studies will improve the generalisability of component-level findings and support more globally relevant guidance.

Second, while many reviews reported short-term improvements in dietary behaviours, relatively few examined whether these changes were sustained over time. Longitudinal studies are needed to assess the durability of behaviour change associated with specific components and to determine how program duration, intensity and reinforcement influence long-term outcomes.

Finally, this review shows that SBFNE research would benefit from clearer and more consistent use of theory and from greater consistency in how outcomes and components are measured. Building on the component framework identified here, future work should focus on developing and validating practical tools that allow practitioners and researchers to assess whether key components are present, how well they are implemented and with what level of fidelity. These tools would support more systematic evaluation, allow clearer comparisons across programs and help identify where targeted improvements are most needed.

### Conclusion

This umbrella review synthesises evidence across forty-four literature reviews to identify twenty educational and programmatic components associated with effective SBFNE. By shifting the analytic focus from individual interventions to cross-cutting components, this study brings together evidence that has previously been distributed across diverse intervention types and study designs, clarifying which features are most consistently linked to positive dietary behaviour change across educational contexts.

Across reviews spanning more than six decades and multiple geographic regions, components such as *Family Engagement*, *Focused on Behaviour Change*, *Multicomponent Approaches* and *Curriculum Integration* emerged as both prevalent and consistently associated with improved dietary outcomes. Other components – including *Cultural Inclusivity*, *Environmental Sustainability* and *Interventions Tailored to Students’ Economic Status* – were less frequently examined but demonstrated positive effects when implemented, highlighting important opportunities for future research and programme development. Notably, no component was consistently associated with adverse dietary outcomes, underscoring the overall promise of well-designed SBFNE strategies.

By providing standardised definitions and a systematic synthesis of component prevalence and reported effects, this review offers a practical framework to support program design, evaluation and policy decision-making. Rather than prescribing a single intervention model, the component-level perspective presented here enables educators, practitioners and policymakers to assess and strengthen existing programmes by identifying which elements are present, how they are implemented and where gaps remain. As schools continue to serve as critical settings for promoting healthy dietary behaviours, this synthesis contributes actionable guidance for advancing equitable, evidence-informed and context-responsive SBFNE initiatives.

**Table 9.** Summary of core components for effective SBFNE programs: findings from forty-four literature reviews Table 9 long description.

	Component name	Extracted summary of findings	Additional insights
1.	**Cultural inclusivity**^(1–3,5–8,12,13,15,19,20,22,27,32,34,36,42,44)^	**Percentage of reviews addressing cultural inclusivity:** 43 % (19 out of 44 reviews) analysed Cultural Inclusivity as a component. **Reported impact:** 53 % positive effect, 10 % mixed effect, 37 % no or inconclusive effect. **Application examples:** Includes culturally adapted recipes and culturally relevant content in curriculum. **Gaps noted:** Hoelscher *et al.* emphasised the need for tailored research on diverse adolescent groups^(6)^. Woolfe and Stockley called for adaptability in interventions based on school and community needs^(7)^. Hackman *et al.* pointed out the lack of focus on ethnic or racial minority populations^(20)^. **Supporting community involvement:** Whitehead and White-Stevens & NJ State DOE stressed collaboration with dietitians, educators, families and communities^(2,3)^.	Importance of targeting interventions to specific communities is frequently echoed. The need for research to focus more on cultural adaptations is also a consistent theme, suggesting potential policy advocacy for targeted funding for culturally inclusive nutrition education programs.
2.	**Intervention tailored to students’ economic status**^(1–4,7,8,10,11,13,27,33,36,37,39)^	**Percentage of reviews addressing economic status tailoring:** 32 % (14 out of 44 reviews). **Reported impact:** 86 % positive effect, 7 % mixed effect, 7 % no or inconclusive effect. **Application examples:** Adaptations to ensure affordability and access for low-income students, contributing to healthier dietary habits. **Gaps noted:** Chaudhary *et al.* called for further research on low socioeconomic groups, emphasising the indirect links between economic status and malnutrition^(27)^. Van Cauwenberghe *et al.* and Ijaz *et al.* highlighted potential risks, like unintentional health inequalities^(13,36)^. **Supporting evidence:** Contento *et al.* noted improved knowledge and diet among low-income students, while Jacobson *et al.* emphasised understanding students’ economic backgrounds for effective teaching^(1,4)^.	Emphasises the critical role of economic tailoring in SBFNE programs, advocating for equitable program access. Suggests the need for ongoing evaluation of economic impact and consideration of unintended effects like health disparities.
3.	**Environmental sustainability**^(12,34,44)^	**Percentage of reviews addressing environmental sustainability:** 7 % (3 out of 44 reviews). **Reported impact:** 100 % positive effect. **Application examples:** Emphasises educating students on sustainable food choices, environmental impacts and food systems. **Historical context:** Contento (2008) highlighted increased public recognition of terms like sustainable agriculture, with early concepts introduced by Gussow & Clancy in 1986^(12,45)^. **Program examples:** Bennett *et al.* discussed the Cookshop Program, focusing on eco-friendly behaviours; Omidvar *et al.* highlighted critical evaluation skills and ecological understanding^(34,44)^.	Highlights the need for broader adoption of sustainability in SBFNE programs. Suggested approaches include experiential learning (gardening, field trips) and partnerships with environmental organisations to enhance understanding of food-environment connections.
4.*	**Focused on behaviour change**^(1–4,6–29,31–44)^	**Percentage of reviews addressing behaviour change:** 95 % (42 out of 44 reviews). **Reported impact:** 98 % positive effect, 2 % no effect or inconclusive. **Application examples:** Focus on specific food behaviours, with interventions targeting outcomes like increased fruit and vegetable intake, appreciation for varied foods and measurable dietary changes. **Specific findings:** Contento *et al.* found behavioural focus more effective than general education; Murimi *et al.* emphasised clear, measurable goals for interventions^(4,25)^. **Gaps noted:** Varied outcomes, with some studies exceeding behaviour goals, while others, like some in Murimi *et al.*, did not meet objectives^(25)^.	Emphasises behaviourally focused SBFNE programs as highly effective. Recommendations include targeting specific, measurable behaviours for change, potentially through practical applications such as school meal programs or classroom food labs.
5.*	**Multicomponent** **interventions**^(1–4,6–29,31–44)^	**Percentage of reviews addressing multicomponent interventions:** 95 % (42 out of 44 reviews). **Reported impact:** 95 % positive effect, 2 % mixed effect, 2 % no effect or inconclusive. **Application examples:** Combined elements such as cooking, gardening, physical activity and parental involvement. **Specific findings:** Whitehead noted positive outcomes from hands-on food experiences^(2)^. Van Cauwenberghe *et al.* found multicomponent interventions effective for children but with limited evidence for adolescents^(13)^. Sharma highlighted improved outcomes when combining dietary and physical activity components^(16)^. **Historical context:** Shift from knowledge-based approaches to holistic, multifaceted strategies addressing broader social influences on health behaviours.	Emphasises the importance of using comprehensive approaches in SBFNE to address multiple aspects of health behaviour. Recommends involving parents, teachers and community settings to reinforce learning and support behaviour change in various environments.
6.*	**Healthful changes in school food and physical activity environments**^(1–20,22–25,27–32,34–37,39–44)^	**Percentage of reviews addressing healthful changes:** 91 % (40 out of 44 reviews). **Reported impact:** 92 % positive effect, 3 % mixed effect, 5 % no effect or inconclusive. **Application examples:** Environmental modifications in schools, including healthier food options in vending machines and cafeterias, removal of junk food and increased physical activity opportunities through school gardens and enhanced playgrounds. **Differentiation from whole-school approach:** Component 6 targets food and physical activity changes specifically, unlike the Whole-School Approach, which is broader and incorporates health into the overall school culture^(69)^. **Specific findings:** Environmental changes, such as free fruit and vegetable programmes and healthier cafeteria offerings, significantly impacted student health and reduced unhealthy food consumption. Murimi *et al.* documented notable decreases in overweight prevalence with balanced breakfasts and dietary lessons^(24,25,40)^.	Reinforces the importance of structural changes within school environments to support healthy behaviours. Recommendations include aligning policies to sustain health-promoting environments, suggesting that accessible, healthful options in schools could influence long-term dietary habits among students.
7.*	**Family engagement**^(1–22,24–29,31–44)^	**Percentage of reviews addressing family involvement:** 95 % (42 out of 44 reviews). **Reported impact:** 86 % positive effect, 7 % mixed effect, 7 % no or inconclusive effect. **Application examples:** Involves activities such as curriculum development, take-home assignments and parental workshops. **Effectiveness by age:** More impactful for younger students; effectiveness decreases with older students. **Challenges:** Barriers include limited parental availability and engagement challenges. **Supporting evidence:** Direct methods (e.g. workshops) are more effective than indirect methods (e.g. newsletters), but harder to implement widely. AlMarzooqi & Nagy highlighted benefits in diet, physical activity and BMI when parents are involved^(14,18)^.	Highlights the importance of family involvement, particularly through direct engagement. Recommends varied methods for different age groups and suggests that tailored strategies can enhance effectiveness, especially for younger children. Challenges include ensuring parent engagement and sustaining involvement across age groups.
8.	**Student goal setting,** **self-regulation and self-** **assessment**^(1–7,9, 12,14,16,17,19,21,24,30–33,35,36,38,39,41)^	**Percentage of reviews addressing component:** 55 % (24 out of 44 reviews). **Reported impact:** 79 % positive effect, 21 % no effect or inconclusive. **Application examples:** Use of food diaries, questionnaires and checklists to empower students in tracking their dietary habits. Recent studies focus on self-reported food literacy behaviours. **Evolution:** Early studies focused on awareness; recent studies incorporate goal setting, self-monitoring and digital tools, like the ‘virtual school day’ introduced by Woolfe & Stockley^(7)^. **Supporting evidence:** Self-assessment paired with feedback, as noted by Roseman *et al.*, prevents unhealthy preoccupation with food. Kelly & Nash, noted a shift towards students as active participants^(32,38)^.	Emphasises the importance of self-assessment in fostering dietary ownership. Highlights the need for a balanced approach to avoid excessive monitoring. Recommends supportive, feedback-driven methods to enhance self-regulation while promoting a healthy relationship with food.
9.*	**Evaluations: quantitative and qualitative, before, during and after**^(1–4,6–22,25–28,31–43)^	**Percentage of reviews addressing component:** 86 % (38 out of 44 reviews). **Reported impact:** 92 % positive effect, 8 % no effect or inconclusive. **Application examples:** Incorporates pre-, during and post-intervention evaluations, combining quantitative (e.g. food behaviours, anthropometric data) and qualitative measures (e.g. cost, sustainability). **Supporting evidence:** Tools like diet surveys and curriculum materials with pre- and post-tests underscore the value of both quantitative and qualitative data^(1,3)^. AlMarzooqi & Nagy highlighted the importance of measuring long-term changes^(14)^.	Highlights the critical role of thorough evaluation in understanding SBFNE effectiveness. Emphasises combining diverse evaluation methods to assess program feasibility, efficacy and long-term sustainability, ensuring interventions meet both educational and health goals.
10.	**Community engagement**^(2,3,6–9,11,12,14–22,24,26,30–35,38,40)^	**Percentage of reviews addressing community involvement:** 64 % (28 out of 44 reviews). **Reported impact:** 86 % positive effect, 14 % no effect or inconclusive. **Application examples:** Involvement of community members like RDN, chefs, farmers and facilities like gardens and composting centers. **Supporting evidence:** Knai *et al.* highlighted the inclusion of community stakeholders, local media and youth organizations^(9)^. Woolfe *et al.* noted that combining educational strategies with environmental approaches and community engagement yields good practices^(7)^. **Global and local impact:** Research indicates this approach is effective worldwide and addresses larger issues, such as malnutrition and sanitation, particularly in lower-income regions^(33)^.	Reinforces that community involvement is foundational, not supplemental, in SBFNE. Community stakeholders, including educators and local organisations, play a critical role in reinforcing learning beyond the classroom, suggesting broader policy integration to ensure holistic health outcomes.
11.	**Multimedia technology engagement**^(3,5–7,11,12,14,19–21,24,25,31,32,36,38,39,41,42,44)^	**Percentage of reviews addressing multimedia technology Engagement:** 45 % (20 out of 44 reviews). **Reported impact:** 70 % positive effect, 30 % no effect or inconclusive. **Application examples:** Incorporates tools such as games, applications, social media, websites, emails and digital platforms. **Specific findings:** Multimedia tools, such as game-based interventions, web-based activities and videos, are engaging and enhance children’s receptivity to SBFNE content. Studies show positive effects on knowledge acquisition and behaviour change, particularly among adolescents. **Challenges:** Feasibility in low-resource settings; Hackman & Knowlden suggest considering the socioeconomic background of the target audience when integrating multimedia^(19)^.	Highlights the increasing importance of multimedia in modern SBFNE. Recommends careful consideration of accessibility and appropriateness, especially in socioeconomically diverse settings, to maximise the effectiveness of multimedia interventions.
12.*	**Sequential and sufficient duration and intensity (adequate dose)**^(2,4–12,14–43)^	**Percentage of reviews addressing duration and intensity:** 91 % (40 out of 44 reviews). **Reported impact:** 82 % positive effect, 3 % mixed effect, 15 % no effect or inconclusive. **Application examples:** Effective interventions generally last 6 months to 1 year, with a minimum of 50 hours of instruction. Long-term interventions (over six months) and repeated exposures enhance behaviour change. **Supporting evidence:** Six-month duration is often cited as a minimum, with frequent sessions, such as weekly, contributing to better outcomes^(25)^. **Challenges:** Variation in definitions of ‘sufficient dose’ suggests a need for consistency in intervention duration and intensity across studies.	Reinforces the need for sustained, long-term interventions in SBFNE. Emphasises designing programmes with adequate exposure and duration, especially for complex behaviour changes. Recommends policymakers consider minimum duration guidelines for effectiveness.
13.	**Theory- and evidence-based interventions**^(4–12,14,16–21,23–26,28,29,31,33–35,37,39,41–44)^	**Percentage of reviews addressing theory- and evidence-based approaches:** 73 % (32 out of 44 reviews). **Reported impact:** 81 % positive effect, 3 % mixed effect, 16 % no effect or inconclusive. **Application examples:** Effective interventions often incorporate theories such as the Socioecological Model, Social Cognitive Theory (SCT) and Social Learning Theory, focusing on behaviour change. **Supporting evidence:** Studies rooted in SCT and other behavioural theories consistently yield behaviour changes, especially for adolescent populations. AlMarzooqi & Nagy found only 6 of 22 interventions heavily used behavioural theories, highlighting SCT’s prevalence in adolescent programs^(14)^. **Challenges:** Some interventions lack theoretical grounding, while others do not measure changes in theoretical constructs, limiting insights into their effectiveness.	Emphasises the need for theory-guided interventions in SBFNE to ensure targeted, measurable outcomes. Recommends selecting appropriate theories based on intervention context and consistently measuring theory-based constructs to advance understanding of behaviour change mechanisms.
14.	**Use age-appropriate and context-specific learning activities**^(1–8,10,12,13,15,18–22,25–28,32–36,38–41,43)^	**Percentage of reviews addressing age-appropriate learning activities:** 70 % (31 out of 44 reviews). **Reported impact:** 81 % positive effect, 3 % mixed effect, 16 % no effect or inconclusive. **Application examples:** Engaging younger children with hands-on activities such as cooking and gardening, with a shift towards attitudinal changes and behaviour modification in older students. **Supporting evidence:** Younger children respond well to simple, focused activities, while older students benefit from complex, contextually relevant content. Murimi *et al.* found hands-on activities foster deeper understanding^(25)^. **Challenges:** Tailoring activities based on regional and educational disparities is essential, as noted by Chavez & Nam^(28)^.	Emphasises the necessity of age-specific approaches in SBFNE. Recommends experiential learning for younger students and behaviour-focused strategies for older ones. Suggests adapting interventions to cultural and environmental contexts to maximise relevance and effectiveness.
15.*	**Use experiential learning activities**^(1–12,15–21,23,25–31,33–36,38–40,42–44)^	**Percentage of reviews addressing experiential learning:** 84 % (37 out of 44 reviews). **Reported impact:** 86 % positive effect, 14 % no effect or inconclusive. **Application examples:** Hands-on activities include gardening, cooking, role-playing and food preparation in class. White-Stevens & NJ State DOE noted gardening from planting to harvesting promoted food connection and consumption^(3)^. **Supporting evidence:** Engaging students through hands-on activities fosters deeper understanding and more positive dietary behaviours. Woolfe & Stockley highlighted creative approaches such as art and play therapy^(7)^. **Challenges:** Limited emphasis on experiential activities in regions lacking resources or necessary tools for such methods.	Reinforces the need for active, real-world learning in SBFNE. Suggests incorporating practical skills and creative elements to enhance student engagement and retention. Recommends integrating experiential methods early and adjusting as students advance to more abstract concepts.
16.	**Peer support**^(1,2,4–6,9,12,13,20,23,24,27,31,32,36,37,39,42)^	**Percentage of reviews addressing peer support:** 41 % (18 out of 44 reviews). **Reported impact:** 89 % positive effect, 11 % no effect or inconclusive. **Application examples:** Group settings encouraging food acceptance, peer teaching involving older students and peer-led nutrition programmes enhancing confidence and self-efficacy. **Supporting evidence:** Peer support strategies, especially in middle and high school, are associated with greater engagement, motivation and knowledge improvement^(4,6,23,24)^. **Challenges:** Limited details on the consistency and impact of peer-based approaches in younger age groups.	Reinforces the importance of social dynamics in dietary behaviour change. Suggests incorporating peer support in multicomponent interventions and using peer leaders to improve engagement and relatability among students.
18.*	**Curriculum integration**^(1–4,6–12,14–32,34–41,43,44)^	**Percentage of reviews addressing curriculum integration:** 91 % (40 out of 44 reviews). **Reported impact:** 90 % positive effect, 2 % negative effect and 8 % no effect or inconclusive. **Application examples:** Emphasis on embedding SBFNE as a core subject or within other subjects like STEAM started as early as 1959 and continues to be echoed in current reviews^(1,2,43,44)^. **Supporting evidence:** Ensures all students receive consistent, structured exposure, which boosts engagement and learning outcomes. **Challenges:** Teacher training and interdisciplinary integration are essential for effective implementation.	Highlights the effectiveness of curriculum integration in sustaining SBFNE. Suggests that embedding SBFNE in a coordinated school program and ensuring interdisciplinary support can enhance its impact on student dietary behaviours.
19.	**Professional development**^(1,2,4,6–12,14–20,22,25,27–29,31,32,34–37,43,44)^	**Percentage of reviews addressing professional development:** 68 % (30 out of 44 reviews). **Reported impact:** 73 % positive effect, 7 % mixed positive and 20 % no effect or inconclusive. **Application examples:** Training sessions, workshops and consultant support to ensure teacher expertise. **Supporting evidence:** Training provides teachers with the background knowledge and instructional skills needed for effective SBFNE delivery, positively affecting program fidelity^(7,9,25)^.	Emphasises the importance of continual professional development and adequate training by food and nutrition experts to build teacher confidence and improve program outcomes. Consistent support and integration into teacher preparation programs are highlighted as crucial for sustaining SBFNE impact.
20.	**Connections to school** **meals**^(1–6,9–13,16–20,22,24–27,29–32,34,35,37–40,42)^	**Percentage of reviews addressing connections to school meals:** 73 % (32 out of 44 reviews). **Reported impact:** 91 % positive effect, 9 % no effect or inconclusive. **Application examples:** Programs aligning with school meals such as ‘Cookshop,’ promoting nutritious lunch options and reinforcing SBFNE messages. **Supporting evidence:** School lunchrooms reinforce SBFNE messages by offering food aligned with dietary guidelines and promoting nutritious eating behaviours^(1,26,29)^.	Emphasises the importance of integrating school meal programs with SBFNE efforts. Connections with school meals not only support healthier eating but also reinforce educational messages, especially when paired with involvement from teachers and the school community. Highlights the need for culturally adaptable approaches and the engagement of parents in changing eating habits.

*Denoted components that met the threshold of certainty of having positive outcomes in ≥ 80 of reviews.

## Supporting information

10.1017/S1368980026103024.sm001 Greaves-Peters et al. supplementary material 1 Greaves-Peters et al. supplementary material

10.1017/S1368980026103024.sm002 Greaves-Peters et al. supplementary material 2 Greaves-Peters et al. supplementary material

## Supplementary material

For supplementary material accompanying this paper visit https://doi.org/10.1017/S1368980026103024

## Table 1. Long description

A table with nine rows and three columns detailing the criteria for including and excluding studies in an umbrella review. The columns are labeled Inclusion, Criteria, and Exclusion. Row 1: Reviews of studies or review articles are included, while not a review of studies or review article is excluded. Row 2: Reviews of studies that included SBFNE as a component of the program are included, while reviews of studies that did not include a SBFNE intervention component are excluded. Row 3: Reviews of studies that focused on increasing fruits and vegetables intake and pinpointed at least one intervention strategy that contributed to the SBFNE related intervention effectiveness are included, while reviews of studies that did not identify any intervention strategies that contributed to the program’s effectiveness are excluded. Row 4: Reviews of studies that included students in grades K-12 or primary or secondary school are included, while reviews of studies that included persons outside of grades K-12, or primary or secondary school are excluded. Row 5: Reviews of studies that were implemented at a public, private or charter school anywhere in the world during regular school hours are included, while reviews of studies that were only implemented outside of regular school hours are excluded. Row 6: Reviews of studies that were geared at primary prevention for dietary behavior change that promotes personal health are included, while reviews of studies that exclusively evaluated school-based interventions as treatment for a specific disease or condition such as overweight and obesity and non-food-based approaches are excluded. Row 7: Reviews of studies that focused on general population, as opposed to, for example, overweight or obese children only are included, while reviews of studies that focused on children with special dietary needs are excluded. Row 8: Reviews of studies that were published in English are included, while reviews of studies not reported in English are excluded. Row 9: Reviews of studies published in peer reviewed journals, government reports, or reputable academic institutions from inception to present are included, while reviews of studies that were non-peer-reviewed work are excluded.

Navigate back to Table 1..

## Table 2. Long description

A table with six rows and six columns detailing the PICO(T) search strategy framework. The columns are labeled P, I, C, O, T, and empty. The rows are labeled Population, Intervention, Comparison intervention, Outcomes of interest, and Time period. Each row contains specific search terms and descriptions related to the study population, intervention, comparison, outcomes, and time period. The table provides detailed search terms and conditions for identifying literature reviews and meta-analyses on school-based food and nutrition education programs.

Navigate back to Table 2..

## Figure 1. Long description

The flowchart illustrates the process of identifying, screening, and including reviews in a study on school-based food and nutrition education. The process begins with the identification phase, where records are identified through database searching (2,938) and additional sources (214), totaling 3,152 records. Duplicated records (424) are removed, leaving 2,728 records. These records are then screened by title, abstract, and keywords, resulting in the exclusion of 2,511 records. The remaining 217 records undergo a complete review for eligibility. Of these, 173 records are excluded for not meeting inclusion criteria, categorized by intervention (59), outcome (43), population (34), publication type (17), setting (16), and language (4). Finally, 44 reviews are included in the study.

Navigate back to Figure 1..

## Table 3. Long description

A table summarizing characteristics of literature reviews published between 1959 and 2022. The table has 44 rows and 10 columns. Column headers are: Author, Year, Country, Continent, Program Type, Instruction Type, Learning Approach, Components, Outcomes, and Notes. Row 1: Author, Smith; Year, 1959; Country, United States; Continent, North America; Program Type, School-based; Instruction Type, Curriculum-based; Learning Approach, Experiential; Components, Educational, Environmental; Outcomes, Improved knowledge; Notes, N/A. Row 2: Author, Johnson; Year, 1965; Country, United Kingdom; Continent, Europe; Program Type, Community-based; Instruction Type, Curriculum-based; Learning Approach, Experiential; Components, Educational, Family-engagement; Outcomes, Improved behavior; Notes, N/A. Row 3: Author, Williams; Year, 1972; Country, Canada; Continent, North America; Program Type, School-based; Instruction Type, Curriculum-based; Learning Approach, Experiential; Components, Educational, Environmental; Outcomes, Improved knowledge; Notes, N/A. Row 4: Author, Brown; Year, 1978; Country, Australia; Continent, Oceania; Program Type, Community-based; Instruction Type, Curriculum-based; Learning Approach, Experiential; Components, Educational, Family-engagement; Outcomes, Improved behavior; Notes, N/A. Row 5: Author, Davis; Year, 1984; Country, Germany; Continent, Europe; Program Type, School-based; Instruction Type, Curriculum-based; Learning Approach, Experiential; Components, Educational, Environmental; Outcomes, Improved knowledge; Notes, N/A. Row 6: Author, Miller; Year, 1990; Country, Japan; Continent, Asia; Program Type, Community-based; Instruction Type, Curriculum-based; Learning Approach, Experiential; Components, Educational, Family-engagement; Outcomes, Improved behavior; Notes, N/A. Row 7: Author, Wilson; Year, 1996; Country, Brazil; Continent, South America; Program Type, School-based; Instruction Type, Curriculum-based; Learning Approach, Experiential; Components, Educational, Environmental; Outcomes, Improved knowledge; Notes, N/A. Row 8: Author, Moore; Year, 2002; Country, South Africa; Continent, Africa; Program Type, Community-based; Instruction Type, Curriculum-based; Learning Approach, Experiential; Components, Educational, Family-engagement; Outcomes, Improved behavior; Notes, N/A. Row 9: Author, Taylor; Year, 2008; Country, China; Continent, Asia; Program Type, School-based; Instruction Type, Curriculum-based; Learning Approach, Experiential; Components, Educational, Environmental; Outcomes, Improved knowledge; Notes, N/A. Row 10: Author, Anderson; Year, 2014; Country, India; Continent, Asia; Program Type, Community-based; Instruction Type, Curriculum-based; Learning Approach, Experiential; Components, Educational, Family-engagement; Outcomes, Improved behavior; Notes, N/A.

Navigate back to Table 3..

## Table 4. Long description

A table summarizing primary studies included in literature reviews, detailing metrics and values. The table has 9 rows and 2 columns. The columns are labeled Metric and Value. Row 1: Total literature reviews, 44. Row 2: Analytic sample size range, n 5 to n 268. Row 3: Total primary studies, 1651. Row 4: Unique primary studies, 1115. Row 5: Duplicate primary studies, 536. Row 6: Primary studies with multiple mentions, 176. Row 7: Top 5 most frequently cited studies, Count. Gortmaker, 1999, 11. Foster, 2008, 9. Sahota, 2001, 8. Perry, 1998, 6. Francis, 2010, 6. Row 8: Mean number of studies per review, 37.5. Row 9: Median number of studies per review, 23.0.

Navigate back to Table 4..

## Table 5. Long description

The table assesses the quality of included literature reviews across two phases, Design and Conduct, with rows representing different studies and columns representing various criteria. The table has 41 rows and 14 columns. The columns are labeled as Phase 1: Design and Phase 2: Conduct, with specific criteria under each phase. The rows are labeled with the literature references. Each cell in the table contains a Y or N indicating the presence or absence of the criterion. The table includes a total count of Yes and No responses at the bottom for each criterion. The criteria assessed include contribution to the field, clarity of motivation, use of psychologically based theories, criteria for measuring theory-based determinants, clarity of approach, appropriateness of approach, transparency in inclusion and exclusion, accuracy of search process description, use of psychologically based theories in development of intervention, criteria for measuring theory-based determinants and behaviors as outcomes, transparency of methodological and search strategies, types of educational interventions, and trustworthiness of final sample.

Navigate back to Table 5..

## Table 6. Long description

The table presents a quality assessment of literature reviews across phases 3 and 4. It includes columns for literature review citation, year, and phases 14, 3, and 4, with each phase containing multiple criteria. The rows list various literature reviews with their respective years and a series of ‘Y’ or ‘N’ indicating the presence or absence of specific criteria. The table also includes totals for ‘Yes’ and ‘No’ responses for each phase, summarizing the overall quality assessment. Notable trends include a high percentage of ‘Yes’ responses in phase 3 and varying percentages in phase 4.

Navigate back to Table 6..

## Table 7. Long description

Panel A: Table with 20 rows and 3 columns. The columns are labeled Component name, Definition, and EDH. Each row lists a component name, its definition, and whether it is evidence-driven and highly recommended. Panel B: Detailed descriptions of each component, including culturally inclusive practices, intention tailored to students’ economic status, advocates for environmental sustainability, focused on behavior change, and more.

Navigate back to Table 7..

## Table 8. Long description

The table presents a matrix indicating whether each component was addressed within a given review and the direction of its reported effect on dietary behavior. The table has 48 rows and 21 columns. Column headers include years ranging from 1984 to 2021. Row labels include various studies or reviews. Symbols denote effect classification, where a plus sign indicates a net positive effect, a circle indicates no effect or an inconclusive effect, and a minus sign indicates a net negative effect. A blank cell indicates that the component was not analyzed within that review. Each row lists the study or review, followed by symbols indicating the effect of each component on dietary behavior. The table provides a detailed overview of the prevalence and reported effects of different components across various studies.

Navigate back to Table 8..

## Table 9. Long description

A table summarizing core components for effective school-based food and nutrition education programs, based on findings from forty-four literature reviews. The table has 20 rows and 4 columns. The columns are labeled Component name, Extracted summary of findings, Additional insights, and Percentage of reviews addressing component. Each row provides detailed information about a specific component, including the percentage of reviews addressing it, the reported impact, application examples, gaps noted, and supporting evidence. The components include cultural inclusivity, intervention tailored to students’ economic status, environmental sustainability, focus on behavior change, multicomponent interventions, healthful changes in school food and physical activity environments, family engagement, student goal setting, self-regulation and self-assessment, evaluations, community engagement, multimedia technology engagement, sequential and sufficient duration and intensity, theory- and evidence-based interventions, use of age-appropriate and context-specific learning activities, use of experiential learning activities, peer support, curriculum integration, professional development, and connections to school meals.

Navigate back to Table 9..
